# Apparatus to investigate the insulation impedance and accelerated life-testing of neural interfaces

**DOI:** 10.1088/1741-2552/aadeac

**Published:** 2018-10-31

**Authors:** N Donaldson, C Lamont, A Shah Idil, M Mentink, T Perkins

**Affiliations:** 1 Implanted Devices Group, University College London, Gower Street, London, WC1E 6BT, United Kingdom; 2 Urest Ltd, 18 St Cross Street, London, EC1N 8UN, United Kingdom; n.donaldson@ucl.ac.uk

**Keywords:** life-testing, encapsulation, integrated circuit, apparatus, accelerated tests, electrochemical impedance spectroscopy, impedance spectroscopy

## Abstract

*Objective*. Neural interfaces and other implantable micro-devices that use polymer-encapsulated integrated circuits will only be allowed in medical devices when their lifetimes can be estimated from experimental data. An apparatus has been developed and tested that allows hundreds of insulated samples (interdigitated combs) to be aged under accelerated conditions of high temperature and voltage stress. Occasionally, aging is paused while the sample’s impedance is measured; the impedance spectrogram may show degradation as it progresses before failure. *Approach*. The design was based on practical considerations which are reviewed. A *Solartron Modulab* provides the frequency response analyser and the femtoammeter. The apparatus can accommodate batches of samples at several temperatures and with different aging voltage waveforms. It is important to understand features of the spectra that are not due to comb–comb leakage, but come from other places (for example substrate-solution leakage); some have been observed and investigated using SPICE. *Main results*. The design is described in detail and test results show that it is capable of making measurements over long periods, at least up to 67 °C. Despite the size of the apparatus, background capacitance is about 1 pF and comb–comb capacitances of about 30 pF can be measured down to 10 mHz, an impedance of about 100 GΩ. An important discovery was the advantage of grounding the bathing solution, primarily in that it raises the measurement ceiling. Observation and SPICE simulation shows that leakage from the substrate to the bathing solution can give phase lags  >90°, in contrast to comb–comb leakage which reduces phase lag to  <90°. *Significance*. The value of this paper is that it will facilitate research into the endurance of small implanted devices because, given a description of a proven apparatus, researchers can start building their own apparatus relatively quickly and with confidence.

## 1. Introduction

Almost all electronic implants rely on polymer encapsulation to prevent leakage currents and corrosion. As implants have become smaller, electric field strengths have increased and yet far less research is done into the choice of materials and methods for encapsulation than into the electronic design. In addition to there being a wide choice of commercially-available materials, researchers would like to be able to test experimental formulations, compare methods of pre-encapsulation cleaning, and compare methods of encapsulation. How do these affect the lifetime of the device in simulated implant conditions? Since many samples of each type may be necessary to get statistically significant results, which can then be used to predict life *in vivo*, an apparatus for such experiments must accommodate a large total number of samples. This paper presents one such design.

The search for ways to provide satisfactory long-term insulation in implanted electrical devices is not new. PEK Donaldson published a paper in 1973 [1] in which he compared materials for encapsulating the discrete components of a visual prosthesis. Historically, electrical tests of encapsulants have been of three types: serpentine tracks that are long and thin conductors that detect corrosion from their increased resistance, interdigitated combs (IDCs) that allow insulation impedance or leakage current to be measured, and triple-tracks which combine both ideas [2]. IDCs are probably now the most popular. The interdigitated combs are often described as *interdigitated electrodes*, a term used in the sensors literature where it was introduced by Hermens in 1984 [3], but we prefer *interdigitated combs* because, as fabricated, they are capacitors with charge stored on the combs. Anderson *et al* started using IDCs to compare encapsulants and the effects of contamination due to inadequate cleaning before application of the uncured rubber; salt contamination caused liquid-filled voids to form which may lead to failure [4].

IDC tests of *insulating biomaterials* were performed by Edell *et al* for many years, funded by the US NINDS Program. They published some remarkable results with a few samples which had lasted for up to 12 years at 37 °C and others for over three years at 90 °C [5]. More recent work has been done by several groups using encapsulated IDCs. Tolstosheeva *et al* [6] compared polyimide with Parylene in soak tests lasting six months at 60 °C. They considered low permeability to water vapour as the key protective property of the coating, rather than its ability to remain adherent to the surface and thus prevent void formation. However, Wang *et al* [7] compared silicone (PDMS), epoxy, Parylene and epoxy+Parylene, apparently finding that silicone was superior on their particular substrate. How polymer encapsulants protect devices has been disputed since the earliest implanted devices.

Much early work on insulation testing was done with constant applied voltages (DC). Edell *et al* provided little information about their apparatus, but described it applying constant voltages to *stress* (age) the samples. They made a 384-channel system, not multiplexed, with one current amplifier per sample [8]. DC testing has been largely superseded by electrochemical impedance spectroscopy (EIS), i.e. AC. With a sinusoidal source, phase-sensitive detection can be used, often with a frequency response analyser (FRA). The FRA acts as a bandpass filter with a Q that is equal to *n* [9], the number of integration cycles, allowing excellent interference rejection but with a fundamental compromise between the duration of the measurement and the error due to interference.

The main advantage of EIS is that the impedance spectrum gives information about the sample being measured and how that changes during an endurance experiment. For example, Madani *et al* [10] fitted circuit models to the impedance spectra to detect bulk absorption of water and de-bonding at the polymer-substrate interface. Features of the impedance spectrum can also include electrochemical effects, such as diffusion-limited reactions [11]. The presence of water in voids is indicated by the characteristic ‘constant-phase’ behaviour of the electrode/electrolyte junctions. Given that so much information may be extracted from features of the observed impedance spectra, it is clearly essential that any features that are not related to the IDC sample itself are understood. We show below that features occur that are not due to the insulation failure at the IDC, and if these are not understood there is a danger that the data will be incorrectly interpreted.

In the CANDO project, (Wellcome Trust: Grant 102037), a brain implant is being developed to treat epilepsy by optogenetic methods. The shafts of the optrodes are to be silicon and these support the LEDs, the recording electrodes, tracks and CMOS ASICs. The silicon will have one or two surface passivation layers (~1 *µ*m) on the top (circuit) side under a thicker conformal layer of silicone (~10 *µ*m). Many questions need to be answered in order to inform these design choices and to justify implantation into patients:

• Of several possible combinations of passivation layer and rubber, which have the longest time-to-failure (TTF)?
• What is the aging acceleration due to temperature?
• How does the TTF depend on the applied voltage waveform?
• How does the TTF depend on the duty cycle?

To answer these questions, we required an apparatus that allows about 250 samples to be maintained at elevated temperatures, with one of several ‘aging voltage waveforms’ applied for most of the time, and with occasional measurements to observe failure or changes in the properties due to degradation.

We have put about six person-years into the development. From the start of the project, some difficulties were obvious but we did not expect to foresee them all and indeed, we have learnt much from testing the apparatus, sometimes leading to modifications. We now understand several phenomena that we met and so we can better interpret the recorded impedance spectra. In this paper we describe the apparatus in its current form, validate its performance and demonstrate some phenomena which we explain by simulation. We think that the value of this paper is that it will facilitate research into the endurance of small implanted devices because armed with a description of proven apparatus, researchers can start building their own apparatus relatively quickly and with confidence.

The paper is arranged as follows: section 2 explains the design and describes the apparatus as it is now. There is additional information in the online supplementary data (stacks.iop.org/JNE/15/066034/mmedia), including photographs which show the apparatus and how it is arranged. Section 3 describes capacitance measurements on an IDC, mounted as in the apparatus: the capacitance values are used in the simulations of section 5. Section 4 describes measurements that validate the apparatus. Section 5 presents SPICE simulations that explain several features of the impedance spectra which we have observed experimentally. Section 6 is a discussion and the conclusions are in section 7.

## 2. Design

**Figure 1.**
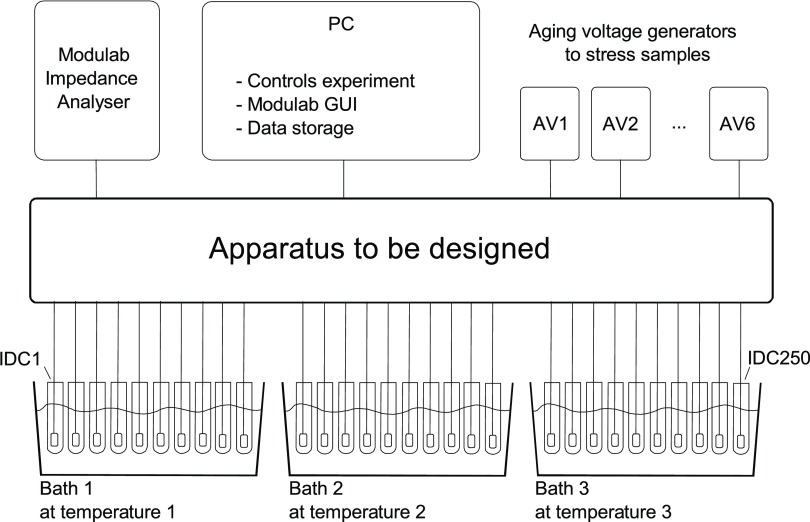
A large number of IDCs are maintained at several elevated temperatures and are electrically stressed by connecting them to one of several voltage sources. Occasionally, each IDC is disconnected from the source and connected to the impedance analyser for measurement.

Figure 1 shows our original concept for the apparatus. For the experiments that we envisaged, we needed about 250 encapsulated IDCs which would be aged more quickly by being held at elevated temperature in simulated body fluid. They would also be electrically stressed by connecting each to one of several voltage sources. From time to time, every IDC would be disconnected from its source and measured with the impedance analyser to obtain an impedance spectrum and eventually to detect failure. At the start of our project, we had to design the apparatus within the central box.

In the following eight subsections of the paper, we have collected technical matters that were relevant to the design.

### 2.1. Design brief

**Figure 2.**
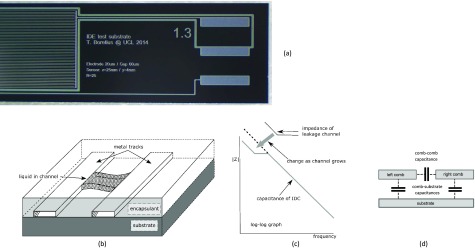
(a) Photograph of part of an IDC showing the bond pads for two combs and the shield. (b) Insulation failure by a channel forming between the combs that fills with condensed water and dissolve any soluble residue. If the channel is 50 *µ*m long, 1 *µ*m high and 100 *µ*m wide filled with pure water (18 MΩ · cm), the resistance of the liquid will be 90 GΩ. The impedance of the electrodes, formed where the channel meets the tracks is in series with this channel resistance. (c) Bode impedance magnitude of the IDC. The magnitude falls at low frequencies when the leakage channel impedance (electrodes  +  liquid) falls below the capacitive reactance of the IDC. (d) Capacitances for IDCs on a conducting substrate.

The simplest model of a fault in an IDC is shown in figure 2: this shows in (a) part of an IDC structure with the bond pads. Such a structure, if perfectly insulated, would behave as small capacitor and so the impedance spectrum will be a line falling at 1 decade/decade as shown in (c). If, however, there is a fault in the passivation or encapsulation so that a void bridges between the metal tracks, this void will contain condensed water that has diffused through the encapsulant. Consequently, in parallel with the small capacitance there will then be a shunt ionic current flowing through the two electrode areas at the ends of the void. The shunt impedance due to this leakage will decrease as the void enlarges, dissolves solutes and perhaps corrosion products: it will appear in the impedance spectrum when its magnitude becomes less than the reactance of the IDC capacitance. The practical resistance limit for detection is therefore given by the formula

1 \begin{align*} \newcommand{\e}{{\rm e}} \displaystyle {{R}_{{\rm lim}}}\leqslant \frac{1}{2\pi \cdot {{f}_{{\rm min}}}\cdot {{C}_{{\rm total}}}}.\nonumber \end{align*}

Where *f*_min_ is the lowest frequency in the spectrum and *C*_total_ is the capacitance of the IDC and additional capacitance from the apparatus itself. For example, if, in figure 2, *C*_total_ is 250 pF, with the void filled with pure water, the change in the slope of the magnitude spectrum will appear at a frequency of 7 mHz (when the reactance of 250 pF is 90 GΩ). The capacitive reactance is the *ceiling* of the measurement because higher shunt impedances cannot be detected. The higher this reactance, or the smaller the capacitance, the better.

Requiring hundreds of samples seemed to have two consequences. First, the apparatus would have to be physically large, perhaps introducing significant extra capacitance in parallel with the IDC. Second, it was important that the time taken to measure each sample was kept short enough for all the samples to be measured at least every few weeks.

The design of the apparatus presented a large challenge, including:

• The susceptibility of measurements to interferences, given the extremely high impedances (up to 1 TΩ) and very small measurement currents (<1 pA);
• The need for the apparatus to be physically large enough to accommodate all the samples, and yet not have such an inherent capacitance which would reduce its sensitivity by bringing down the measurement ceiling;
• The samples must be held at elevated temperatures to accelerate aging;
• The electronics of the apparatus must itself be protected from long-term exposure to moisture; and finally,
• It must be practical to take measurements in experiments lasting years and with reliable data-collection.

Assuming one can only afford one FRA, there is a conflict between apparatus sensitivity (impedance of detectable void) and the number of samples under test. Little has been published about apparatus for life-testing implanted devices by EIS. There are helpful general guidelines from Keithley [12] for high-impedance instrumentation. Troyk [2] has presented some useful principles for soak testing (when the samples are submerged) but when we started the development of the apparatus, we did not know what ceiling could be achieved when so many samples were present, and we also did not know how difficult it would be to prevent interference.

### 2.2. Instrument for EIS

The apparatus utilises a Solartron *Modulab ECS*, a potentiostat with an FRA and femtoammeter. The potentiostat of the *ECS* can be altered to a constant voltage source by connecting the reference electrode (RE) to the counter electrode (CE). This is what we have done in the work described below and consequently the input to the femtoammeter is described as the working electrode (WE) terminal, and the voltage source as the CE terminal.

The femtoammeter [13] extends the measurable current, with 12 ranges, the lowest being 3 pA (full scale) with a resolution of 0.15 fA and an accuracy of 5%. Less sensitive ranges are more accurate. In most of our experiments, the instrument is used for EIS (although in some we apply 0 V and measure the current due to interference effects). The range in use may be fixed or put in *auto* mode: the latter has the advantage that resolution is maximised and random noise from the femtoammeter amplifier is minimised (section 2.4.2 in [13]) but there may be switching artefacts which mar the data [14]. The output data from an EIS test includes time domain samples of applied voltage and measured current. This sample rate is adjustable during set-up but defaults to 2 samples s^−1^. A useful feature of the *Modulab* is an output terminal from the femtoammeter so that an oscilloscope can be used to see the noise and interference which is present in addition to the sinusoidal current coherent with the drive voltage at CE.

### 2.3. Time to measure each spectrum

For EIS, the applied frequency can be extended to the *µ*Hz range but this is impractical; tests would take too long, and we have never worked below 1 mHz. The FRA generates the sinusoidal source voltage at CE. The experimenter sets the number of decades of frequency, the number of frequencies per decade, and the amplitude. Integration occurs over an integer number of cycles which may be fixed or be determined from the convergence of the average integrals (real and imaginary) according to the set-up.

EIS measurements are usually made at a predetermined number of frequencies per decade. If that number is *k* and the spectrum is measured from *f*_max_ down to *f*_min_, also supposing that at each frequency the FRA integrates over c cycles, then the total time will be (geometric series):

2 \begin{align*} \newcommand{\e}{{\rm e}} \displaystyle T=\frac{c\left(\frac{1}{{{f}_{\min}}}-\frac{1}{{{f}_{\max}}\sqrt[k]{10}} \right)}{1-\frac{1}{\sqrt[k]{10}}}.\nonumber \end{align*}

For example, if *k*  =  5, which gives a good impression of the shape of spectra, and the frequency changes from 100 kHz to 0.01 Hz with *c*  =  3 cycles per frequency, then the total time is 811 s, close to 13½ min, of which 300 s, 5 min, is at the lowest frequency. Thus the total time is dominated by the lowest frequencies.

### 2.4. Effect of conductive substrate

Another consideration is the effect of the substrate of the IDC. A formula was given by den Otter [15] for the capacitance between the combs in an infinite volume of dielectric. This formula can be modified for the case where the IDC lies in the plane between semi-infinite volumes of dielectric 1 and dielectric 2.

3 \begin{align*} \newcommand{\e}{{\rm e}} \displaystyle C=\frac{2A{{\varepsilon}_{0}}}{\pi a}\left({{\varepsilon}_{{\rm R}1}}+{{\varepsilon}_{{\rm R}2}} \right)\sum\limits_{n=1}^{\infty}{\frac{1}{\left(2n-1 \right)}J_{0}^{2}\left(\frac{\left(2n-1 \right)\pi s}{2a} \right)}.\nonumber \end{align*}

*A* is the area of the IDC, *s* is the gap between fingers and *a* is the finger width plus the gap. For the IDC shown in figure 2(a), *A* is 1 cm^2^, *s* is 60 *µ*m and *a* is 80 *µ*m. Suppose that the substrate is quartz (silica, ${{\varepsilon}_{{{R}_{{\rm 1}}}}}$  =  4.2) and there is no coating so ${{\varepsilon}_{{{R}_{{\rm 2}}}}}$  =1 (air). The series in equation (3) converges quickly and taking the first ten terms of the Bessel function, the calculated capacitance, the comb–comb capacitance between the combs, is 23 pF. However, we want to test CMOS structures with metal combs over a conductive silicon substrate. Using the parallel plate capacitor formula for the area of one comb and with a SiO_2_ dielectric layer (*ε*_R_  =  3.8) of 1 *µ*m thickness, the calculated comb-substrate capacitance is 420 pF (figure 2(d). The comb–comb capacitance is much smaller than the comb-substrate capacitances and if the substrate is floating and the capacitance is measured between the combs, the apparent capacitance will be (420/2)  +  23 pF, due to the two comb-substrate capacitances being in series. The presence of the conducting substrate has increased the apparent capacitance and therefore lowered the ceiling of the leakage measurement.

### 2.5. Design concepts

We started by assuming that we would have some switches between the *Modulab* and the IDCs; also that the best switches would be reed relays. We tested several types of reed relay and chose the Coto 2341-05-010 because it had the highest leakage resistances across the open contacts, >2 TΩ when activated and  >20 TΩ when not activated. They require 5 V and 22 mA through the solenoid to activate. However, when we made a small printed circuit board (PCB) with three reed relays connecting the *Modulab* to one of three IDCs, we found that if we used a mains power supply to provide the coil currents, the measurements were spoilt, whereas with a floating battery, very high impedances could be measured. The risk of electrical interference from other mains-connected sources also led us to choose optical fibres for the channel-select signals.

**Figure 3.**
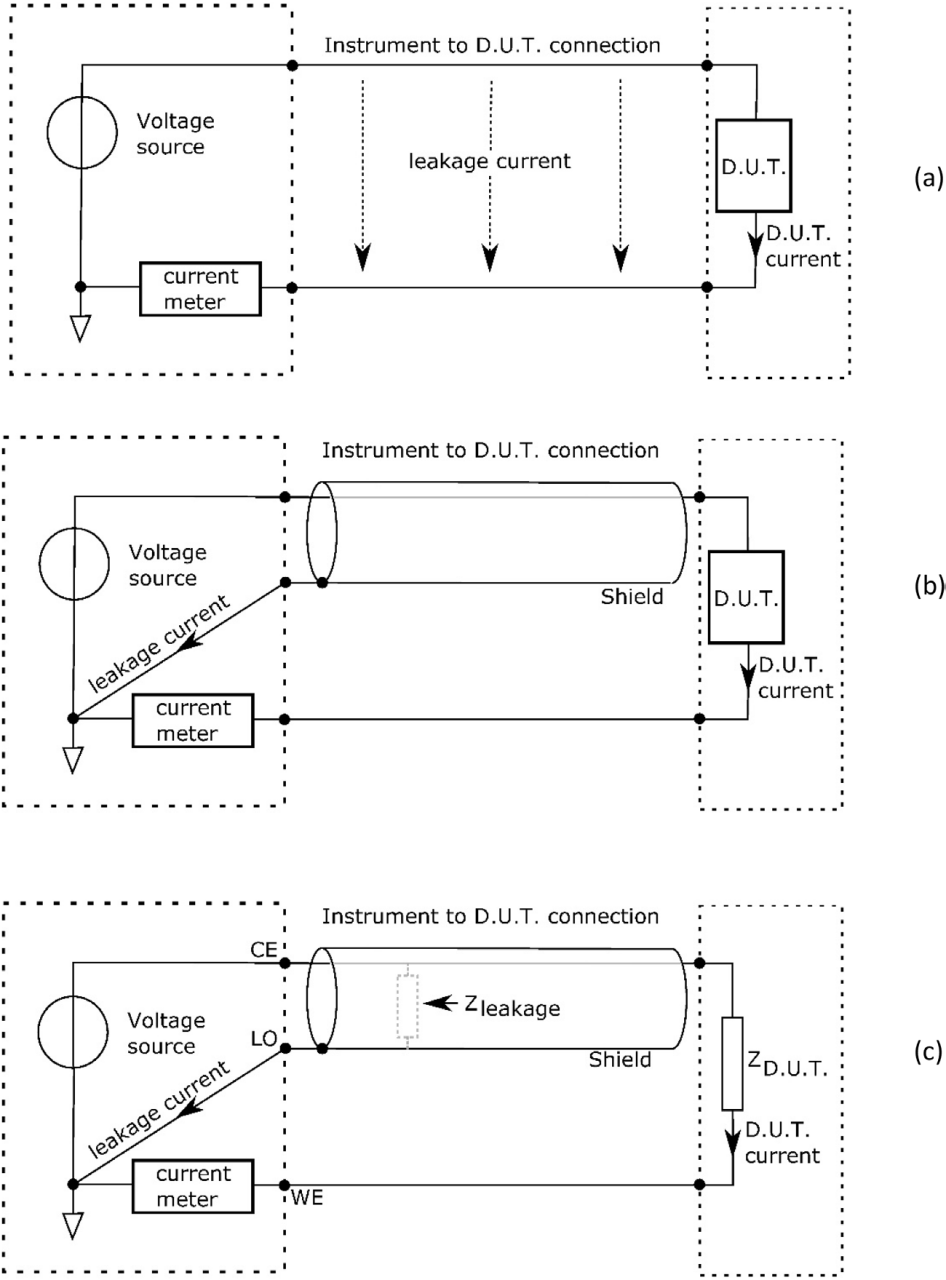
(a) Measurement of the impedance of the device under test (D.U.T.), inaccurate because leakage current and device current both flow to the ammeter. (b) Shield conductor is added so that leakage current by-passes the ammeter. (c) In AC measurements using a potentiostat (with RE connected to CE), the ammeter measures the current through *Z*_DUT_ while the voltage source also supplies current to the leakage impedance.

*Shielding* is a well-known method that is shown in figure 3. In the three diagrams, the *Modulab* is represented by the voltage source and the ammeter on the left. In figure 2(a), the ammeter measures the sum of the current in the DUT and the leakage. The shield conductor, shown in figure 2(b), lies between the source track (CE) and the ammeter track (WE), intercepting the leakage current and carrying it to ground bypassing the ammeter. Shields are mostly tracks on surfaces that collect surface currents. In AC measurements, the leakage can be represented by an impedance from CE to LO, as shown in figure 2(c). In our design, the shield must be carried all the way along the WE conductor from the IDC back to the *Modulab*. Shields are also sometimes called *guards*. In this paper we use the word *screen* to mean an electrostatic (earthed) sheet to limit an electric field in space. In passing, note that the impedance measured in EIS is the ratio of the source voltage to the ammeter current in figure 3(c).

Like Edell, we decided that each sample should be in a separate container so that they would be electrically isolated (but see sections 2.8 and 4.6). Specifically, that each should be in a tall test tube, containing the IDC in phosphate-buffered saline (PBS), heated at the bottom and cooled at the top to discourage evaporation. Unlike Edell, we decided to use hot water baths with thermostats for heating, instead of heated metal blocks with holes into which tubes are inserted. That choice meant that we have to make up evaporation losses from the bath water but it ensured good heat conduction to the PBS so that we could be confident about the applied temperature. We found large hot-water baths (SAP5, Sub Aqua Pro, Grant Instruments) that would just fit on our benches and that could contain an array of 96 one-inch diameter test tubes in 14 rows, with seven tubes in each row, except the two end rows, with only six (figure 6(a)).

**Figure 4.**
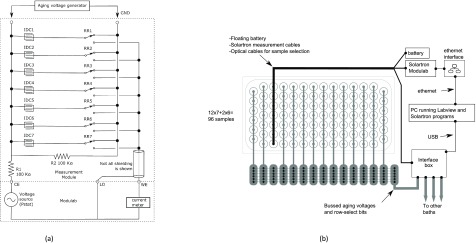
(a) Multiplexer that allows either impedance measurement of one IDC or application of the aging voltage to all IDCs in parallel. Note that during impedance measurement, all the other six IDCs are connected in parallel to *R*_2_, so negligible voltage is then applied across them. *R*_1_ limits the current in case any sample fails as a short-circuit. (b) Overview of one tank with 14 rows of tubes, 13 with the usual connection via the backplane to the Interface box. The third row is shown during measurement with its temporary connections.

If it takes about 20 min to measure the EIS of one sample down to 0.01 Hz (section 2.3), then with seven samples in a row, the theoretical shortest time to measure them all would be 2 h and 20 min. In practice, we often use a measurement mode that integrates for longer in order to improve the accuracy (in the presence of intermittent interference) resulting in increased experiment time, approaching half a day. We assumed that the seven samples in one row would be selected by seven reed relays, but the question arose whether another bank of relays should automatically select the row within the tank [2]. We decided not to do this because it would have made the electrical system even more complicated and perhaps difficult to understand. Given that we could expect to take half a day for each row, we preferred to make a semi-automatic system where the experimenter manually disconnects one row (*module*) from the *backplane* and connects it instead to the *Modulab* for half a day of impedance measurement. This is shown in figure 4: (a) being the simplified circuit for one row, and (b) the block diagram for the complete system (but with only one of several tanks). In figure 4(a), either the *aging voltage* is applied to all seven IDCs in parallel or the impedances are measured one by one.

### 2.6. The IDC, the adaptor and the bung

Figure 2(a) shows the end of an IDC. There are three pads, WE, SH and CE from the top. The shield track can be seen surrounding the IDCs (except at the other end where the CE track joins its comb).

**Figure 5.**
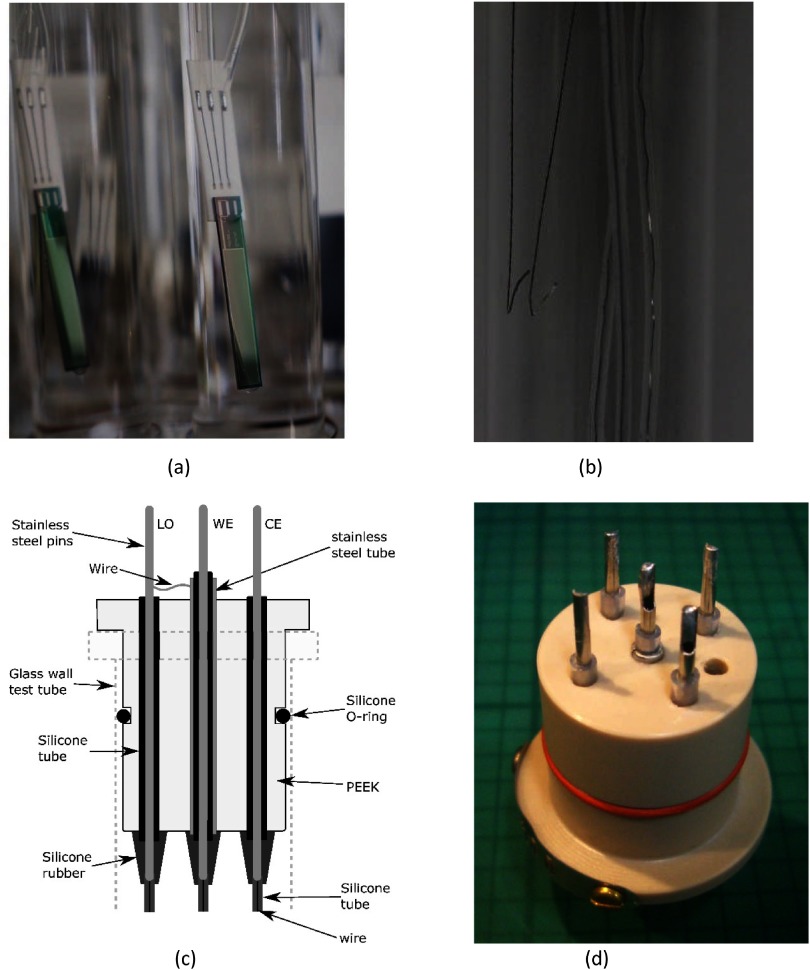
(a) IDC glued to ceramic adaptor with gold wire bonds to platinum–gold thick-film tracks and solder joints to stainless steel wire. The wire is insulated with PTFE. A second layer of insulation is the 1 mm OD silicone tube around each wire. The silicone tube is glued to the ceramic. The IDC and the adaptor are then dip-coated with heat-cured 2-part silicone rubber. (b) There are *depth electrodes*, bared wires, to detect the level of the PBS. (c) Cross-section through the bung shows three of the five stainless steel pins that pass through the bung, forming a connector above. Underneath, the wires are soldered to the pins and the joints encapsulated with RTV silicone. By having silicone tubes passing through the PEEK, adhesion from RTV to PEEK is unnecessary. (d) Bottom view of bung before wires are soldered to the pins. There is a tapped vertical hole closed by a nylon screw to allow topping up of the liquid without removing the bung from the test tube.

Figure 5 shows how the WE conductor is shielded as it passes through the bung by a concentric tube that is connected to shield (LO). There is also a SH pin for a wire that goes to the shield on the adaptor and thence the IDC.

Below the bung, where the water vapour pressure is high, good insulation should be possible because the RTV silicone can remain bonded to the silicone tube for long periods in the presence of water, and so each conductor is intubated from the adaptor to the top of the bung. The RTV must be applied carefully so that there are no leakage paths between pins and also so that all metal surfaces (stainless steel and solder) are coated with RTV to prevent corrosion.

The tops of the stainless steel pins are gold-plated so there are gold–gold contacts to reduce the risk of bad connections.

### 2.7. Water management

Despite the fact that the o-ring that seals the bung in the tube is only silicone rubber, at 67 °C the rate of water loss from the tubes is negligible and has not need topping up after one year.

**Figure 6.**
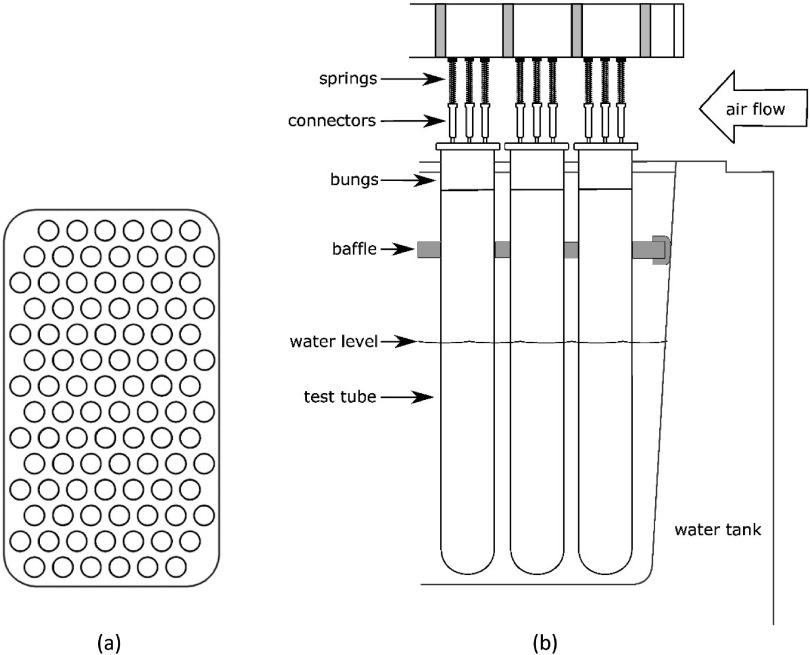
(a) Plan view of 96 tubes in the tank, 12 rows of seven and two rows of six tubes. (b) Diagram showing water management. Evaporation is reduced by the baffle which is a 1.5 mm thick silicone rubber sheet sandwiched between 1.5 mm thick sheets of polycarbonate. There is a rubber gasket made of U-section silicone rubber extrusion which seals the baffle to the wall of the tank. A row of five small fans blow lab air through the space above the bungs and under the measurement modules. Typically, the relative humidity in this space is about 35% (it is warm above the tanks).

To prevent leakage current not at the IDCs, in addition to shielding the WE conductor, it is also desirable to keep PCB surfaces dry. The PCBs are standard FR4 with solder-resist from ITEAD Intelligent Systems Co., not special high-resistivity types, and therefore it is important that water evaporating from the bath does not condense on the PCBs above. Figure 6(b) shows that there is a baffle above the water surface to reduce the rate of water vapour escape and above it, an airway, ventilated by a row of five fans, to carry the vapour out into the lab.

In the tank, there are water-depth and water-temperature sensors; the water has to be topped up every few days. Above the baffle, in the airway, are temperature and relative humidity sensors, all connected to the GUI (see online supplementary data). Usually, the temperature above the baffle is higher than room temperature and the humidity is lower, presumably because of the warmth from the tank.

The PBS in the tubes cannot be seen because of the large number of tubes and the baffle (see figure 6 and supplementary figure C) and therefore some method was required to detect whether the level of the PBS in each tube was getting low. We decided to have two ‘depth electrodes’ in each tube, stainless steel wires with their insulation removed at the ends. The impedance between these bare wires is about 100 Ω at 1 MHz when fully immersed in the PBS.

### 2.8. Measurement module

**Figure 7.**
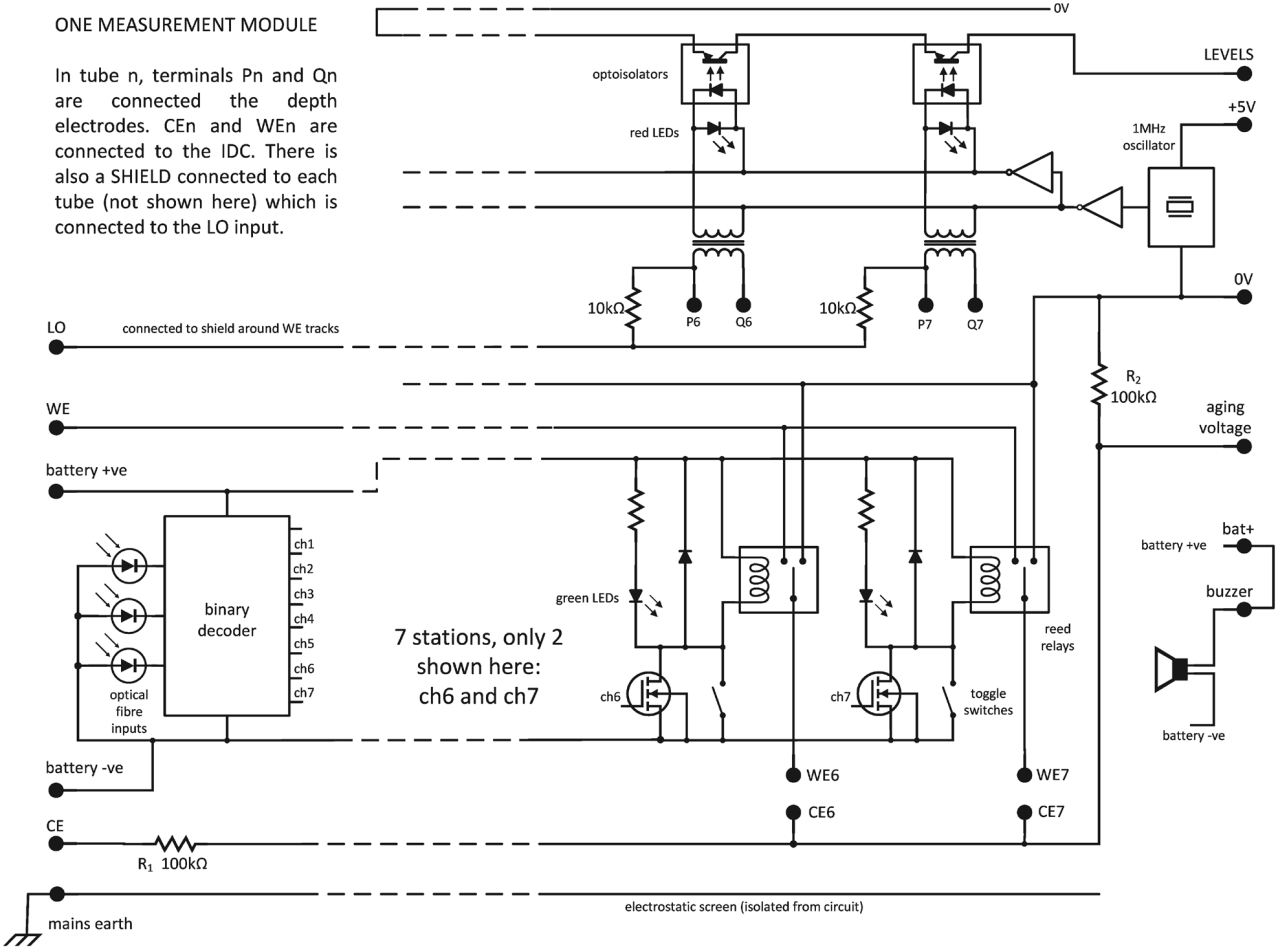
Schematic diagram of one measurement module. Except for ‘mains earth’, all the connections on the left are made only during measurement, and on the right during aging. The permanent earth connection only goes to the metal frame and the perforated metal screen. Each tube has connections WEn, CEn, SHIELDn, Pn & Qn. Pn & Qn are the depth electrode connections to the secondary of the corresponding transformer: their primaries are driven in parallel from the 1 MHz oscillator, and the opto-isolators OR the outputs so if one or more level is low, it can be detected. The light detector diodes for the three fibres are shown on the left. The toggle switches allow the reed relays to be tested without the optical fibre inputs: usually these toggle switches are open.

The complete schematic diagram for the measurement module is shown in figure 7. A photograph of one module is supplementary figure A. The schematic shows the following.

• There is a socket on the left for the floating battery (~6.5 V) used during EIS measurements, to power the module without mains interference.
• There are three fibre-optic receivers (red light) that carry a binary address that is used to select one of the seven channels (0 0 1 to 1 1 1).
• The outputs of the decoder are connected to the gates of seven FETs which, when switched on, activate a reed relay and a parallel green LED showing which tube is selected.
• On the right hand side is a 6-way DIN socket. If a plug is in the socket while the battery is connected, the buzzer sounds (to avoid making bad measurements).
• One side of each transformer secondary (P1–P7) is connected to LO by a 10 kΩ resistor. These were added and are a significant modification. The advantages are described in section 4.6 and the choice of 10 kΩ is justified in section 5.3.
• The 5 V supply in the DIN connection causes the 1 MHz oscillator to excite the seven transformers in parallel which feed the depth electrodes. If the depth electrodes are in PBS, current flows in the primaries and also through the series red LEDs and the opto-isolators. This test is done for a few seconds every hour and not during sample impedance measurement.
• The opto-isolator outputs are connected in series. If the PBS in any tube is low, the corresponding red LED will not light, and the impedance from the output LEVELS will be high. In this way, a row module with one or more depleted tube can be automatically detected and then the particular tube can be identified by looking at the LEDs.
• The sockets for the tubes are mounted below the narrow horizontal PCB (see figure A in the supplementary data).
• On the horizontal PCB, there are shield rings in the copper around the WEn connections on both surfaces.
• The 2 mm stainless pins that project above the test tubes are inserted into gold-plated female connectors (Multi Contact, LS2, 0-B).
• Beryllium–copper helical springs are used to accommodate poorly aligned pins without significant stress. These are soldered to the brass screws in the horizontal board and to the connectors with 60/40 Sn/Pb solder with water-soluble *Hydro-X* flux (Loctite HYDX 60EN), and the joints cleaned soon after soldering by rinsing in tap water.
• The top and bottom of the horizontal PCB, plus the springs and solder joints to the connectors, are coated with adhesive silicone (Dow Corning 3140).
• Each module has a grounded perforated metal screen on one side to reduce capacitive coupling between modules.
• There are two variants of the module (A & B) because of the staggered rows of tubes in the tank (figure 6).

After assembly and electrical testing, the lower half of the module is cleaned in a detergent solution followed by many rinses in water, finishing with 18 MΩ · cm deionised water, then blown dry in nitrogen and baked at 50 °C for 1 h before the silicone is applied to the horizontal board.

The remainder of the hardware and the GUI are described in the online supplementary data.

## 3. Capacitor models of the IDC in the tube

As explained in section 2.4, ideally-insulated IDCs have only a small capacitance between the combs while IDCs over a conductive substrate also have capacitances between each comb and the substrate which may be substantially larger.

**Figure 8.**
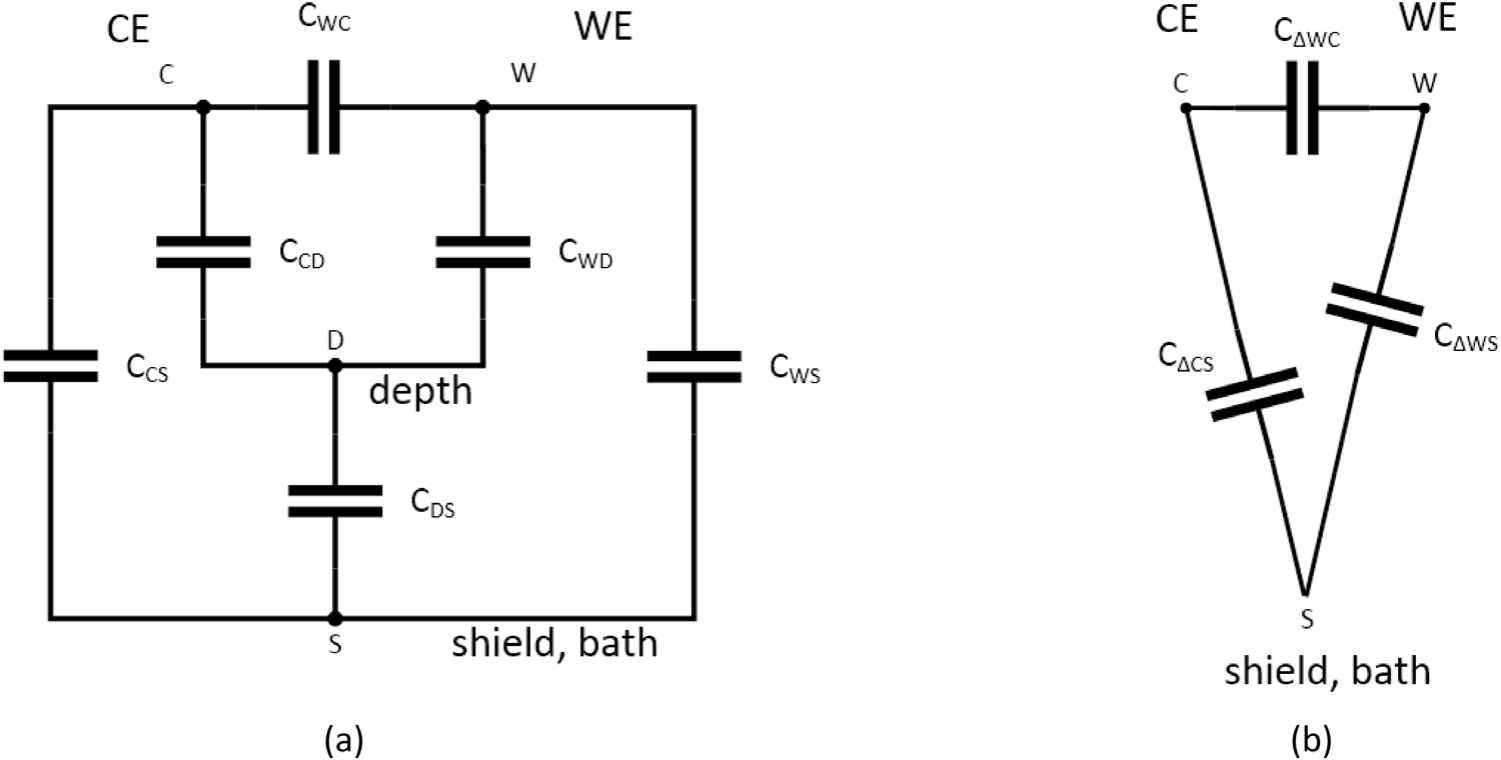
(a) 4-terminal model and (b), 3-terminal delta model of tube. If there is no connection to node D in (a), then the network can be represented by the delta, shown in (b): see text.

In the experiment, the physical structure is much more complicated and yet we need to be able to model it. The IDCs are is in the test tube with its adaptor, wires and bung, all insulated, with PBS and depth electrodes (see figure 5). The tube itself is immersed in the hot water bath which is grounded to mains earth. The tube has five pins for electrical connection but the two for the depth electrodes have a low impedance through the PBS and through the secondary winding of the transformer during measurement (~100 Ω) so we treat them as one in this model. During measurement, the shield is connected to LO on the potentiostat and the bath is connected to mains earth, so these are, at least in principle, at the same potential, and are considered as one node. Thus the model has four nodes (CE, WE, shield/bath, and depth electrodes). As shown in figure 8, this requires six capacitances to represent it before any leakages occur.

We found these six capacitances for a typical sample (figure 2(a)) by making six measurements of capacitance (*C*_1_–*C*_6_) between groups of nodes as shown in table 1, using a hand-held capacitance meter.

**Table 1.** Six capacitance measurements.

Measurement	From nodes	To nodes	Capacitance (pF)
*C*_1_	w	c d s	556
*C*_2_	c	w d s	570
*C*_3_	d	w c s	1349
*C*_4_	s	w c d	315
*C*_5_	w c	d s	1070
*C*_6_	w s	c d	849

Then the matrix equation (4) is solved

4 \begin{align*} \newcommand{\e}{{\rm e}} \displaystyle \left[\begin{array}{@{}cccccccccccccccccccc@{}} 1 &amp; 0 &amp; 0 &amp; 1 &amp; 1 &amp; 0 \nonumber \\ 1 &amp; 1 &amp; 0 &amp; 0 &amp; 0 &amp; 1 \nonumber \\ 0 &amp; 1 &amp; 1 &amp; 0 &amp; 1 &amp; 0 \nonumber \\ 0 &amp; 0 &amp; 1 &amp; 1 &amp; 0 &amp; 1 \nonumber \\ 0 &amp; 1 &amp; 0 &amp; 1 &amp; 1 &amp; 1 \nonumber \\ 1 &amp; 0 &amp; 1 &amp; 0 &amp; 1 &amp; 1 \end{array} \right]\left[\begin{array}{@{}c@{}} {{C}_{{\rm WC}}} \nonumber \\ {{C}_{{\rm CD}}} \nonumber \\ {{C}_{{\rm DS}}} \nonumber \\ {{C}_{{\rm WS}}} \nonumber \\ {{C}_{{\rm WD}}} \nonumber \\ {{C}_{{\rm CS}}} \end{array} \right]=\left[\begin{array}{@{}c@{}} {{C}_{1}} \nonumber \\ {{C}_{2}} \nonumber \\ {{C}_{3}} \nonumber \\ {{C}_{4}} \nonumber \\ {{C}_{5}} \nonumber \\ {{C}_{6}} \end{array} \right].\nonumber \end{align*}

To get the values shown in table 2. These values are used in the simulations of section 5.

**Table 2.** Capacitance values in figure 8(a).

*C*_WC_	*C*_WS_	*C*_CS_	*C*_WD_	*C*_CD_	*C*_DS_
28	11	7	517	535	297

Notice that the capacitances between tracks on the IDC (CE-WE, WE-shield, CE-shield) are all small compared to the capacitances to the depth electrodes.

In the 4-node model of figure 8(a), D is the centre of a star (*C*_CD_, *C*_DS_, *C*_WD_) inside a delta (*C*_WC_, *C*_WS_, *C*_CS_). If the depth electrodes are not connected to any other node, or it is justifiable to neglect this connection (see below) then there are only three nodes on each tube. In that case, only three capacitances are needed to represent it. Using the star-delta transformation, the *three* capacitances of the delta are given by:

5 \begin{align*} \newcommand{\e}{{\rm e}} \displaystyle {{C}_{\Delta {\rm WC}}}={{C}_{{\rm WC}}}+\frac{{{C}_{{\rm CD}}}{{C}_{{\rm WD}}}}{{{C}_{{\rm CD}}}+{{C}_{{\rm WD}}}+{{C}_{{\rm DS}}}}=28+205=233\,{\rm pF}\nonumber \end{align*}

6 \begin{align*} \newcommand{\e}{{\rm e}} \displaystyle {{C}_{\Delta {\rm CS}}}={{C}_{{\rm CS}}}+\frac{{{C}_{{\rm CD}}}{{C}_{{\rm DS}}}}{{{C}_{{\rm CD}}}+{{C}_{{\rm WD}}}+{{C}_{{\rm DS}}}}=7+118=125\,{\rm pF}\nonumber \end{align*}

7 \begin{align*} \newcommand{\e}{{\rm e}} \displaystyle {{C}_{\Delta {\rm WS}}}={{C}_{{\rm WS}}}+\frac{{{C}_{{\rm DS}}}{{C}_{{\rm WD}}}}{{{C}_{{\rm CD}}}+{{C}_{{\rm WD}}}+{{C}_{{\rm DS}}}}=11+114=125\,{\rm pF}{\rm .}\nonumber \end{align*}

These capacitances are used in the simulations that are presented in section 5.

## 4. Validation tests

### 4.1. The effect of placing an IDC sample into the apparatus

**Figure 9.**
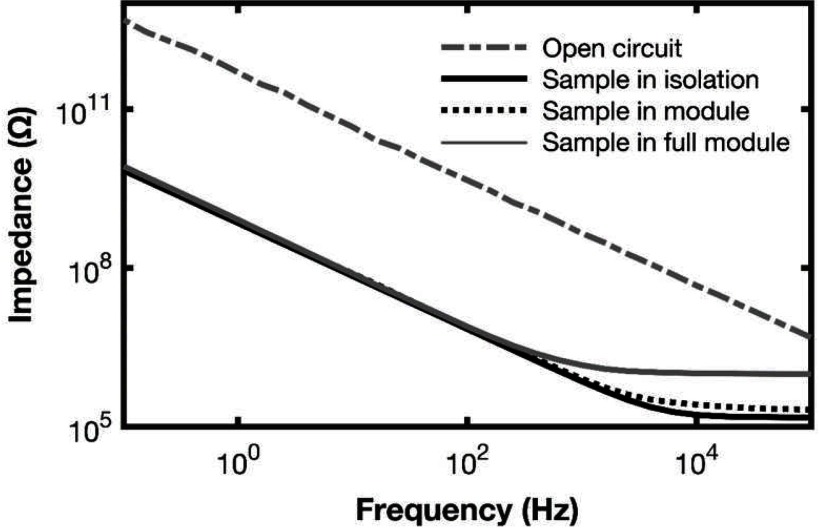
The effect of placing an IDC sample in the apparatus. The low-frequency capacitances are 0.55 and 227 pF.

For baseline impedance measurements, we have a small Faraday cage (metal box) which allows the tube, containing the encapsulated IDC and PBS, to be connected to the *Modulab* while it is in air. Figure 9 shows the impedance without a tube in the Faraday cage, then with the tube in the Faraday cage. That tube was then connected to a module, suspended in water in the tank at 67 °C, and measured again. Finally, six other samples are added to the module and the measurement repeated. The impedance at low frequency is 413 times lower than the open cable. At high frequencies, above 200 Hz, the plateau resistance depends on the number of other tubes connected.

### 4.2. Where is the capacitance that is being measured?

Three samples are compared in this test: (1) an encapsulated adaptor without any IDC attached to it, such that the wires from the bung go only to the adaptor; (2) an IDC sample for which the WE and CE tracks have been ablated by laser between the wire bond pads and the IDC itself (see figure 2) but otherwise encapsulated in the usual way; and finally (3) a complete sample. Their six capacitance values were measured, as described in section 3, and the 4-node model capacitances calculated (table 3). All measurements were at 67 °C.

**Table 3.** Comparison of samples described in section 4.2.

		Δ capacitances			Star capacitances		
	Capacitance (pF)	*C*_WC_	*C*_WS_	*C*_CS_	*C*_WD_	*C*_CD_	*C*_DS_
1	Adapter	0.7	9.9	2.4	9.2	6.7	8.3
2	Laser ablated	0.9	10.9	2.9	46.0	45.6	60.6
3	With IDC	28	11	7	517	535	297

*C*_WS_ is larger than *C*_CS_ in lines 1 and 2 because of the shield tube around the WE pin at the bung. These capacitances are only raised slightly when comparing the adapters to the laser-ablated sample, suggesting the capacitance between the wire bonds and IDC bond pads is negligible.

The main result is that of the 28 pF of *C*_wc_ for the complete sample, only 0.9 pF is not in the IDC itself.

### 4.3. Can we see changes in the IDC impedance with age?

**Figure 10.**
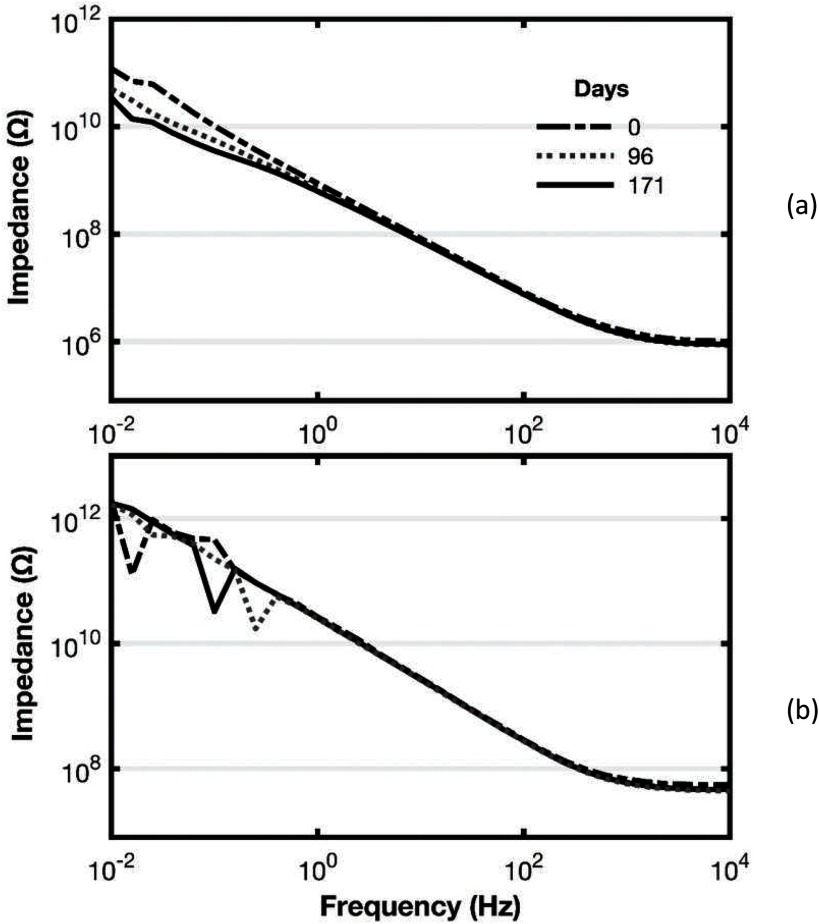
(a) Changes with age seen in a sample over 171 d: the impedance falls by one order of magnitude at 10 mHz. (b) Shows the impedance of an ‘empty tube’ over the same period. In (b), the underlying capacitive characteristic can be seen (and does not change) but there are erratic points: at first we did not know what caused these outliers (but see figure 11).

As an example to illustrate aging, figure 10 shows a sample from our third group (Group C) which have silicon oxy-nitride passivation and Nusil MED3-4013 silicone rubber. This sample was measured over 171 d, submerged at 67 °C. A high duty cycle  ±5 V biphasic aging voltage had been applied for 76% of that time. This waveform is  +5 V for 500 *µ*s, −5 V for 500 *µ*s, then 0 V for 1 ms. We also show measurements over the same period for a tube in the same module which has no adaptor or IDC, and is therefore a blank measurement. Figure 10 shows these impedance magnitude plots. These graphs show that the apparatus is able to detect the expected progressive deterioration in the IDC and that the change is not elsewhere in the module.

Figures 10(a) and (b) have outlying points in the spectra but clearly the background measurement in figure 10(b) is steady over the 171 d with water bath at 67 °C, whereas the IDC shows a fall of about an order of magnitude at low frequencies.

### 4.4. What causes the outlying points in the spectra seen at low frequencies?

**Figure 11.**
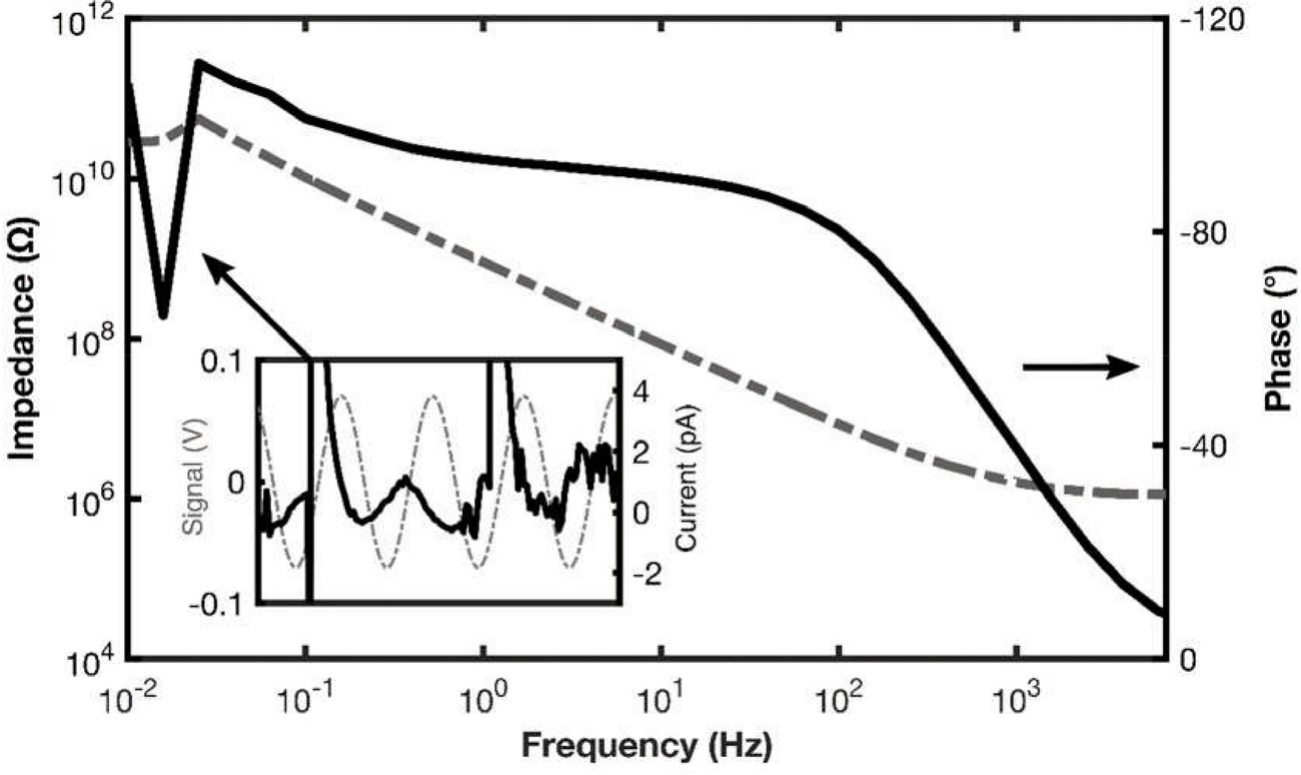
The inset graph shows the sinusoidal voltage at CE and the current from WE. The sinusoidal current, which alone can give valid impedance values, is small compared to two glitches. Their occurrence, while measuring the impedance at about 20 mHz, has caused the outliers in the spectrum.

As mentioned above, the *Modulab* has an output from the femtoammeter which allows the input current to be observed. Its software also allows points in the spectrum, such as one of the erratic points, to be marked and the corresponding point in the frequency sweep (i.e. voltage versus time) to be displayed. Both of these methods allow the interference that causes the erratic points to be inspected. A common cause of the outliers are glitches like that shown in figure 11, where an outlier in the spectrum is associated with two glitches in the femtoammeter current which are much larger than the sinusoidal current due to the sinusoidal source.

These glitches may be infrequent so that, when one occurs at high applied frequencies, the FRA can continue integration until the average phase and quadrature currents have converged. But if the interval between glitches is similar to the period of the applied sine wave, then the average will not converge and an erratic point is produced. That is why the spectra become erratic at low frequencies.

### 4.5. Phase lags  >90°

**Figure 12.**
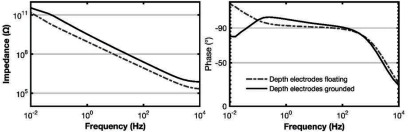
ID047 showed phase lag over 90° at low frequency (dashed curve) while the depth electrodes were only connected to the transformer (see figure 7). The effect of adding the 10 kΩ resistors from one depth electrode to LO is to decrease the maximal lag, although it remains over 90° for about three decades of frequency (continuous curve).

For some samples, we found that the phase at low frequencies did not do what we expected. If there is no leakage, the phase would be expected to be close to 90° of lag at low frequencies, but leakage would decrease the impedance magnitude and decrease the phase lag. What we saw was phase lags over 90°, as illustrated in figure 12 for one sample.

We found that there are several advantages of connecting one of the depth electrodes to LO through a resistor. We chose a value of 10 kΩ, which is explained in section 5.3, and we did this for all tubes, as shown in figure 7. One benefit of doing this is that it curbs the excess phase lag although it does not necessarily eradicate it, as shown by the measured spectrum in figure 12.

### 4.6. Benefits from *grounding the depth electrodes*

**Figure 13.**
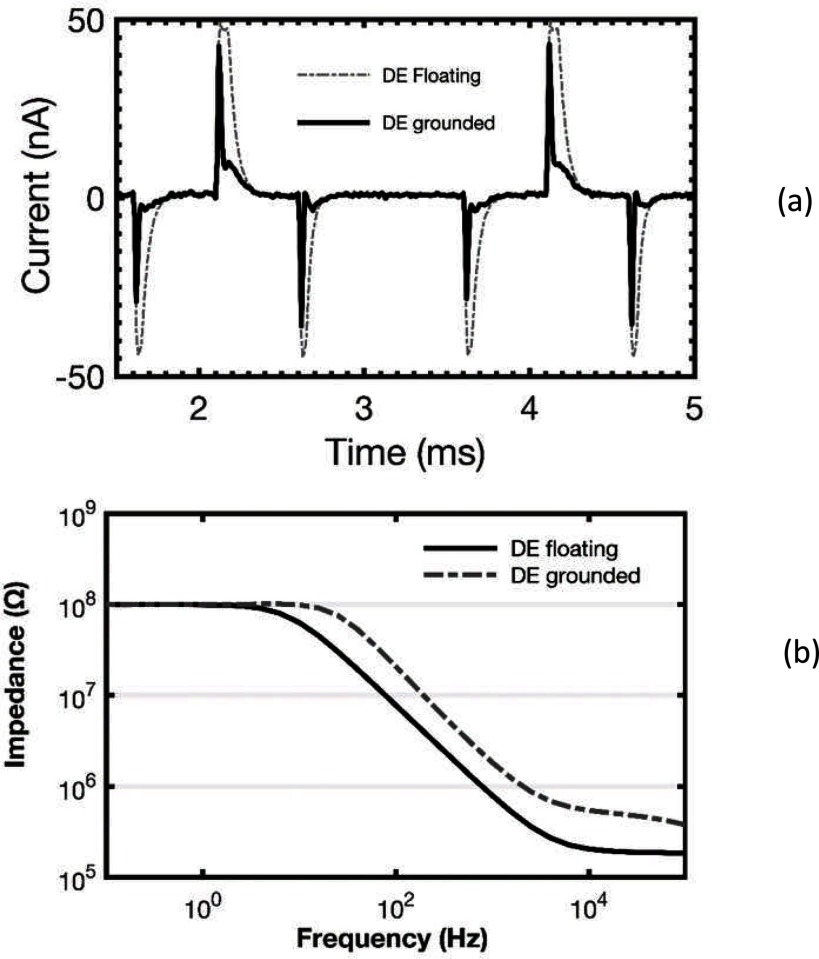
Other beneficial effects of connecting one depth electrode in the tube to LO via a 10 kΩ resistor. (a) Interference is from biphasic aging voltage that is being applied to many other IDCs in the tank. To get this record, the output of the femtoammeter was connected to an oscilloscope. The femtoammeter current range was fixed to prevent automatic range-changing. (b) An IDC sample in a tube has a 100 MΩ resistor connected from CE to WE to imitate a shunt leakage. The graph shows how grounding the depth electrode raised the ceiling but the sensitivity to shunt leakages is not diminished.

The connection of one depth electrode to LO is through a 10 kΩ resistor, which is very small compared to the impedances being measured, so we call this *grounding the depth electrodes*. In addition to reducing the low-frequency phase lag, it has two other advantages. The first is that interference from the aging voltage generators is reduced because the PBS is acting as a screen, though not a very effective one, between the water in the bath and the DUT. The artefact when the aging voltage generator is applied to many other IDCs in the tank is shown in figure 13(a), with and without the 10 kΩ resistor.

The second, more important, advantage is shown in figure 13(b). Grounding the depth electrode raises the ceiling by reducing the measured value of the capacitance to the comb–comb capacitance without the additional comb-substrate capacitances. However, it does not alter the apparent value of leakage resistance. *This useful effect will be explained in* section 5.4.

## 5. Understanding the spectra: SPICE investigation

### 5.1. High-frequency asymptote

**Figure 14.**
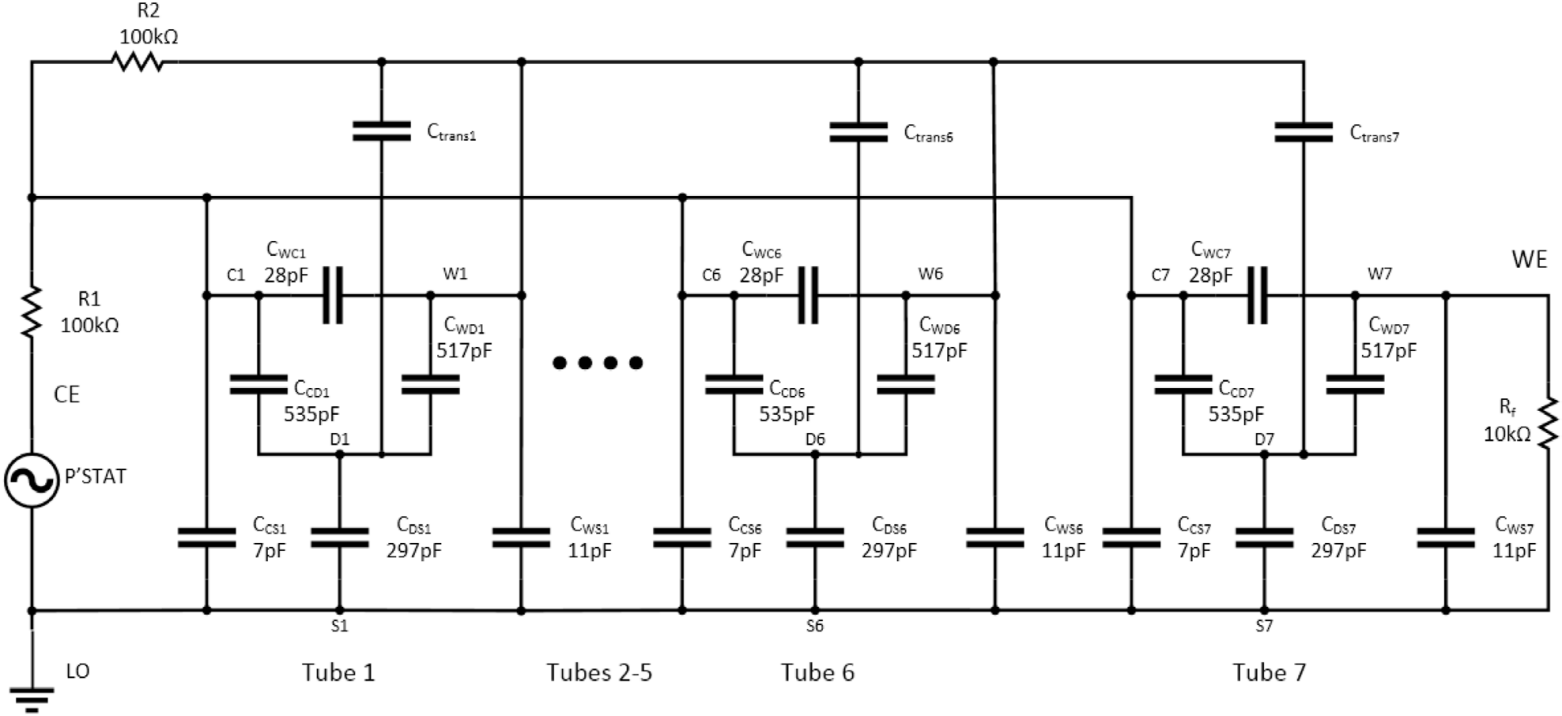
Model of module during measurement, only the 1st, 6th and 7th tubes shown, while the 7th is being measured. The femtoammeter resistance is *R*_f_. *C*_trans_ is the capacitance between the primary and secondary windings of the transformers.

**Figure 15.**
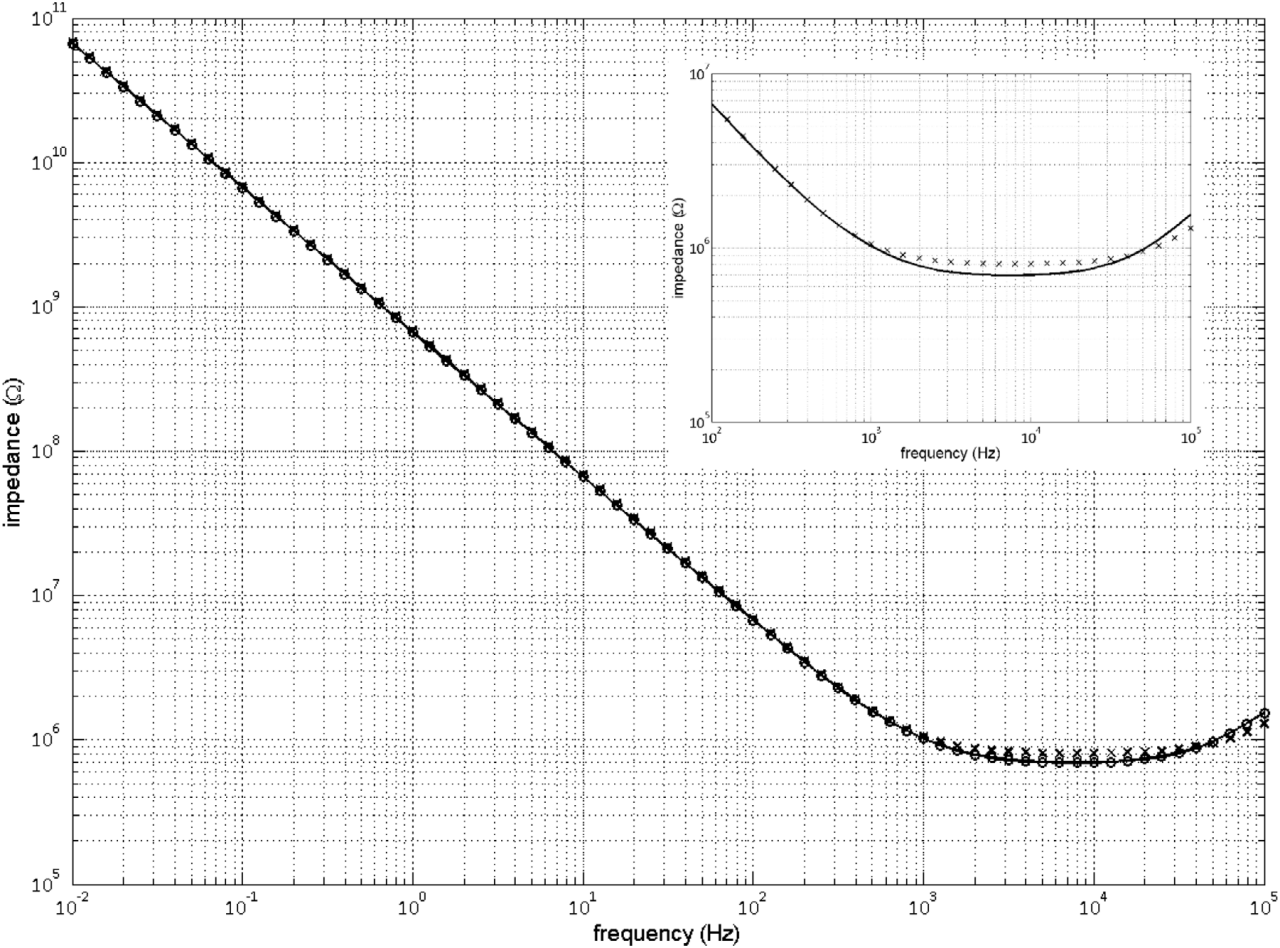
The measured impedance in figures 14 and 16. The impedance for the complete model (line), the double delta model (*o*) and the band-pass filter model (*x*). The difference between the complete model and the band-pass filter are shown inset.

Figure 14 is a circuit diagram for a module with seven tubes, one of which is being measured. The measured impedance of the DUT is the ratio of the source voltage to the femtoammeter (*R*_f_) current. The capacitor values come from table 2. The capacitors C_trans_ are between the primary and secondary of the transformers (measured as 28.4 pF). *R*_1_ and *R*_2_ are the actual resistors shown in figures 4 and 7. A SPICE circuit simulator (LTSpice, Milpitas, CA, USA, version XVII (×64)) was used to investigate this circuit and the circuits in the remainder of this section of the paper. Here it was used to find the magnitude of the measured impedance, the ratio of the potentiostat voltage (*P’stat*) to the current in the femtoammeter (*R*_f_). The Bode magnitude plot is shown in figure 15.

**Figure 16.**
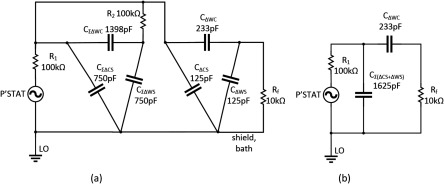
(a) double-delta model; (b) band-pass filter model.

The full circuit of figure 14 can be reduced by the following steps:

• Disconnecting the capacitors *C*_trans_ from the depth electrodes nodes does not alter the impedance transfer function, so they can be removed. As there is then no extraneous connection to the D nodes (depth electrodes), the 4-node model of each tube can be changed to the 3-node model, i.e. from figures 8(a) to (b).
• Then lump together the three capacitances in tubes 1–6 and call them *C*_ΣΔWS_, *C*_ΣΔWC_ and *C*_ΣΔCS_. That gives the double delta model shown in figure 16(a). The transfer function for this simplified circuit is indistinguishable from the original impedance magnitude shown in figure 15.
• Observe that the two components *C*_ΣΔWC_ and *R*_2_ make little difference (the voltage across *C*_ΣΔWC_ is small) so they can be removed.
• Observe that *C*_ΔWS_ in figure 16(a) has little effect, being shunted by the relatively low input impedance of the femtoammeter, so it can also be removed.

That leaves the much simplified circuit shown in figure 16(b), a band-pass filter. *C*_Σ(ΔCS+ΔWS)_ is now (7  ×  *C*_ΔCS_  +  6  ×  *C*_ΔWS_)  =  1625 pF coming from all the IDCs. Figure 15 shows that this is slightly different from the original but it retains the main features. The capacitance at low frequencies (below 100 Hz) is 233 pF (i.e. C_ΔWC_ from the measured IDC)

The transfer function of this network is

8 \begin{align*} \newcommand{\e}{{\rm e}} \displaystyle Z=\frac{{{V}_{{\rm in}}}}{{{I}_{{\rm f}}}}=&amp;\,\left(\frac{1}{j\omega {{C}_{\Delta {\rm WC}}}} \right)+\left({{R}_{1}}+{{R}_{{\rm f}}}+R_1 \frac{{{C}_{\Sigma (\Delta {\rm CS}+\Delta {\rm WS})}}}{{{C}_{\Delta {\rm WC}}}} \right)\nonumber \\ &amp;+\left(\,j\omega {{C}_{\Sigma (\Delta {\rm CS}+\Delta {\rm WS})}}{{R}_{1}}{{R}_{{\rm f}}} \right)\nonumber \end{align*}

which shows the plateau and the rising impedance seen at high frequencies. The plateau impedance is the sum of *R*_1_  =  100 kΩ, *R*_f_  =  10 kΩ and $\left(\frac{{{C}_{\Sigma (\Delta {\rm CS}+\Delta {\rm WS})}}}{{{C}_{\Delta {\rm WC}}}} \right)\cdot {{R}_{1}}$ or (1625/233)  ×  100  =  697 kΩ, a total of 807 kΩ. It is much higher than *R*_1_ alone, which is what it would be if the model were simply the 110 kΩ of *R*_1_  +  *R*_f_ in series with *C*_ΔWC_.

### 5.2. Phase lags  >90° with depth electrodes floating

We expect that the interesting changes that occur due to aging of the samples will be below the first cut-off frequency, about 1 kHz in figure 15. In this region, according to the circuit models of figures 14 and 16, the phase should be lagging at 90°. Greater phase lags, as observed in figure 12, imply a 2-stage filter: how can that occur?

**Figure 17.**
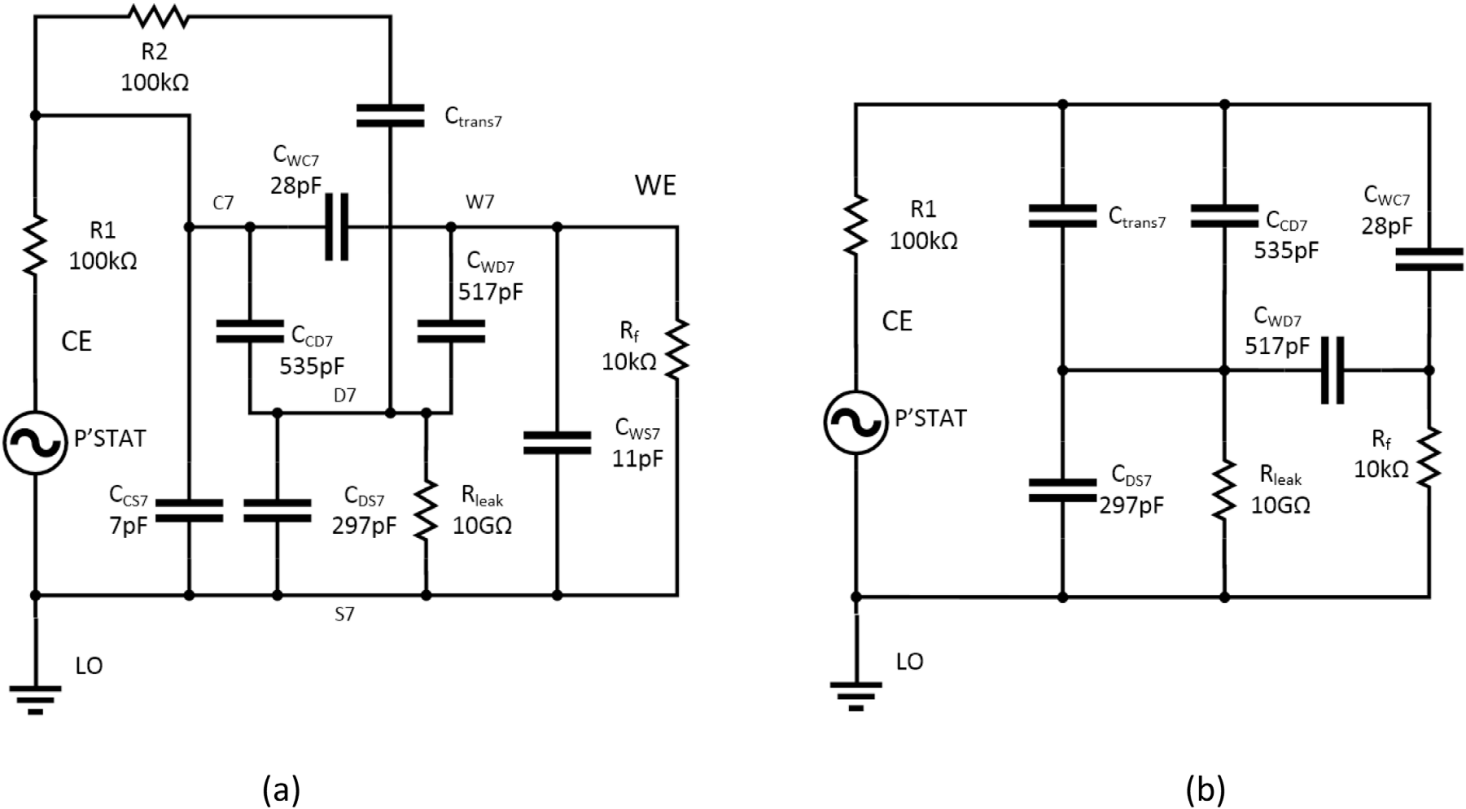
(a) One tube with 10 GΩ leakage resistance from depth electrode; (b) simplified circuit with *R*_2_ shorted and both *C*_WS7_ and *C*_CS7_ deleted. The effect of the leakage resistor can be seen in figure 18.

**Figure 18.**
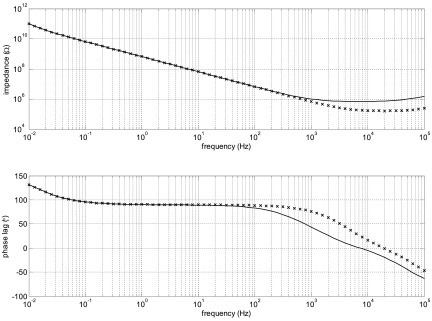
The solid curves show the full model (figure 14) with a 10 GΩ resistor from D7 (one of the depth electrodes) to LO (figure 17(a)). The curves marked by x are for the circuit of figure 17(b), which shows a good fit at the frequencies where the phase lag exceeds 90°.

Looking at figure 14, it can be seen that the impedance at the depth electrodes node D7 is high. If a leakage impedance appears from that node to LO, which could be between adjacent pins on the bung, what effect would it have? This was simulated by adding a 10 GΩ resistor to the model to represent such a leakage (figure 17(a)). Figure 18 shows that this causes a similar lag greater than 90° at the lowest frequencies, reaching 131° at 10 mHz, very like what we observed (see figure 12).

To see why this happens, we again looked for a simpler circuit model. Because we are here only interested in the low frequencies, we omitted the 6 tubes that are not being measured figure 17(a). SPICE simulation showed that neither *C*_CS7_ nor *C*_WS7_ have significant effect. Also there is no voltage drop across *R*_2_ at these frequencies, so it can be shorted, leaving the circuit of figure 17(b). This is a 2-stage filter whose first stage low pass cut-off is at:

9 \begin{align*} \newcommand{\e}{{\rm e}} \displaystyle f=\frac{1}{2\pi {{R}_{{\rm leak}}}\left({{C}_{{\rm trans}7}}+{{C}_{{\rm DS}7}}+{{C}_{{\rm CD}7}} \right)}={\rm 3}\,{\rm mHz}.\nonumber \end{align*}

The phase in the voltages at D7 and therefore at W7 start to increase as the reactance of the three capacitors surpasses the resistance of the leakage resistance.

Such sensitivity to very large leakage resistances is obviously important. Leakages from the depth electrode conductors to the shield conductors might occur in many places: at the bung, either above or below, in the measurement module, or between the depth electrodes and the shield track on the adaptor through the PBS.

Because the effect on the impedance spectrum of such leakage is opposite to the effect of lateral leakage between the combs which we expect to see as the IDC deteriorates, there is a danger that these other leakages from the depth electrodes will confound the results.

### 5.3. Benefit of adding resistors from one depth electrode to LO in each tube

**Figure 19.**
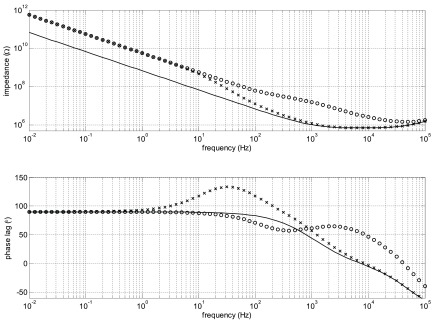
The effect of connecting one depth electrode in every tube to LO through a resistor. The solid line show the impedance with no resistors; the curves marked with *x* are with 1 MΩ resistors; and with o are with 10 kΩ resistors.

Figure 14 represents the original circuit but, as mentioned in section 4.6, we discovered that there are many advantages of connecting one of the depth electrodes in each tube to LO via a resistor, and we chose a value of 10 kΩ. The reason for this choice of resistance is illustrated in figure 19 which was derived from the full model (figure 14 with additional resistors). The measured impedance with no resistors is compared to 1 MΩ resistors and 10 kΩ resistors.

Actually this is the same situation as in figure 18 but here with much lower resistances to LO. The effect of these resistances is to cause a wave in the phase plot which was below 10 mHz when the resistance was 10 GΩ, and appears here at 30 Hz for 1 MΩ and at 3 kHz for 10 kΩ. This wave does not spoil the part of the impedance spectrum in which we are interested, at low frequencies, if the resistance is sufficiently small. 10 kΩ is satisfactory, according to this simulation, because the measured impedance below 100 Hz is of a pure capacitor. We could have made a direct connection but then the impedance would have been unpredictable, depending on the submerged lengths of the bared wires that are the depth electrodes.

One of the advantages of adding these resistors is that leakage from the depth electrode conductors to the shield (LO) are less serious, because they are in consequence in parallel to the low-value resistors. This removes one effect that might otherwise confound the results.

### 5.4. Ceiling

**Figure 20.**
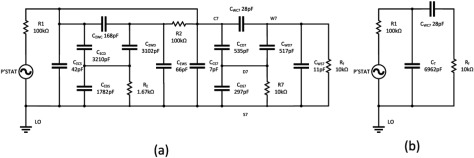
(a) Represents the full model with tubes 1–6 lumped together. (b) Is the reduced model which is a good fit at low frequencies. At high frequencies, the expected resistive plateau for the model in (b) is at $\left(\frac{{{R}_{1}}{{C}_{{\rm T}}}}{{{C}_{{\rm WC}7}}} \right)=25$ MΩ, as shown in figure 21.

A remarkable point about figure 19 is that, due to adding the 10 kΩ resistors, the impedance is increased at low frequencies by a factor of about 8, decreasing the capacitance to 28 pF. To see why this happens, consider the two schematic diagrams in figure 20. Figure 20(a) represents the complete module with the added 10 kΩ resistors but with the first six tubes lumped together. The 28.4 pF capacitances across the transformers have been omitted because they are a very high impedance relative to 10 kΩ.

At low frequencies, the value of all the resistors is so low that they can reasonably be considered short-circuits. It follows that the capacitances *C*_ΣCS_, *C*_ΣCD_, *C*_ΣWD_, *C*_ΣWS_, *C*_CS7_ and *C*_CD7_ can all be combined to a capacitance *C*_T_ of 6962 pF. *C*_WD7_ and *C*_WS7_ can be omitted because they are shunted by the low impedance of the femtoammeter. The capacitance in series with *R*_f_ is only *C*_WC7_ of 28 pF. The reduced circuit is shown in figure 20(b) and should be compared to figure 16(b).

Where *C*_ΔCS_ is found from equation (6), *C*_ΔWS_ is from equation (7) and:

10 \begin{align*} \newcommand{\e}{{\rm e}} \displaystyle {{C}_{{\rm T}}}={{C}_{\Sigma {\rm CS}}}+{{C}_{\Sigma {\rm CD}}}+{{C}_{\Sigma {\rm WD}}}+{{C}_{\Sigma {\rm WS}}}+{{C}_{{\rm CS}7}}+{{C}_{{\rm CD}7}}.\nonumber \end{align*}

Table 4 shows why the ceiling is raised in figure 19 by adding the 10 kΩ resistors: the capacitance that is in series with the femtoammeter (*R*_f_) has been decreased from 233 to 28 pF. This is because the current cannot flow through the substrate via the capacitances between the substrate and the combs. By adding the 10 kΩ resistors, the PBS in the tubes is held at earth potential and that is capacitatively-coupled to the substrate through the thin layer of insulation so that it is also grounded. Consequently, current that flows from CE (i.e. voltage source) to the substrate flows to LO and not to the femtoammeter. Little current flows through the capacitance from the WE comb to LO because that capacitance (*C*_WD7_  +  *C*_WS7_) is shunted by the low impedance of the femtoammeter.

**Table 4.** The effect of grounding the depth electrodes.

	Figure 16(b)		Figure 20(b)	
	Capacitors	Value (pF)	Capacitors	Value (pF)
Series capacitance	*C*_ΔWC_	233	*C*_WC7_	28
Shunt capacitance	*C*_Σ(ΔCS+ΔWS)_ = 7 × *C*_ΔCS_ + 6 × *C*_ΔWS_	1625	*C*_T_	6962

**Figure 21.**
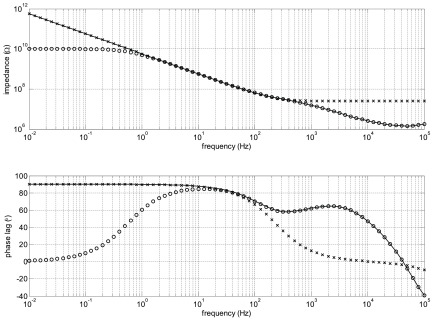
Comparison of the full model (continuous), the 4-component model (*x*) and the effect of a 10 GΩ leakage resistance on the full model (*o*). The full model is well approximated by 4 components at low frequencies. The magnitude at low frequencies represents a capacitance of 28 pF.

The impedance spectra for the full model (figure 14 but with added 10 kΩ resistors) and the 4-component model of figure 20(b) are compared in figure 21. There is a close resemblance below 500 Hz in magnitude and 100 Hz in phase.

This raising of the measurement ceiling by a factor of 8 was an unexpected discovery and we wondered whether it might affect the sensitivity to the leakages between the combs that we want to detect on the IDCs. However, adding a leakage resistance to the full circuit model (figure 14) shows that this is not the case: the third spectrum in figure 21 shows how a 10 GΩ shunt appears as a 10^10^ Ω asymptote at low frequencies. This shows that the addition of the 10 kΩ resistors between on depth electrode and LO in each tube raises the ceiling (background impedance), without altering the sensitivity to the leakage impedance that we are trying to measure.

### 5.5. Phase lags  >90° when depth electrode connected to LO through 10 kΩ

**Figure 22.**
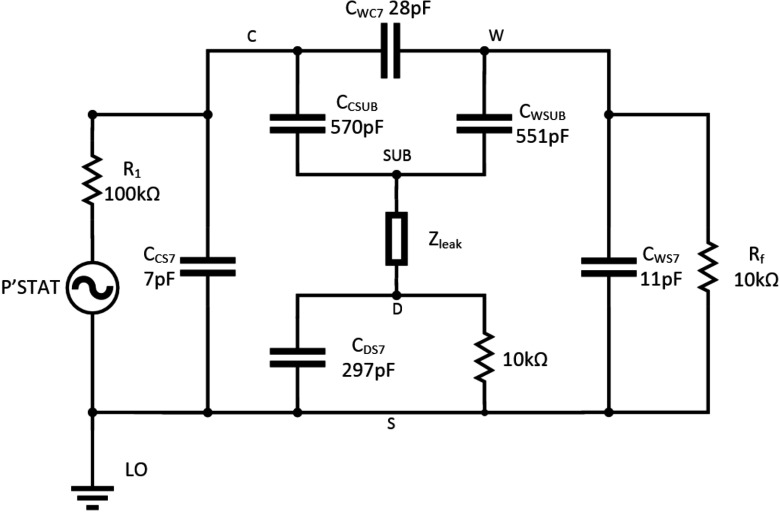
The *SUB* node has been added with a leakage impedance *Z*_leak_ through the encapsulation from the substrate to the PBS.

Figure 12 shows that even when one depth electrode is connected to LO through 10 kΩ, phase lags over 90° may still be observed. As shown in section 5.3, this must still be due to a 2-stage high-pass filter appearing. The circuit model of figure 22 shows how this may occur. The capacitor model for one tube (figure 8(a)) has been elaborated to include a node that is the silicon substrate under the IDCs: instead of just two capacitors, *C*_WD_ and *C*_CD_, there is now an additional impedance *Z*_leak_ between the substrate and the depth electrodes. The values shown in the figure were calculated using the formula for parallel plate capacitors for *C*_wsub_ and *C*_csub_ and the known thickness of the thermal oxide. *Z*_leak_ was measured for one sample (on which, due to an insulation fault, we had an ohmic connection to the substrate) as a capacitance of 8.6 nF at 67 °C. This is much larger than one might expect from the substrate area and the average thickness of the silicone but we suppose that this is due to the very thin silicone covering of the corners of that substrate (which was dip-coated).

**Figure 23.**
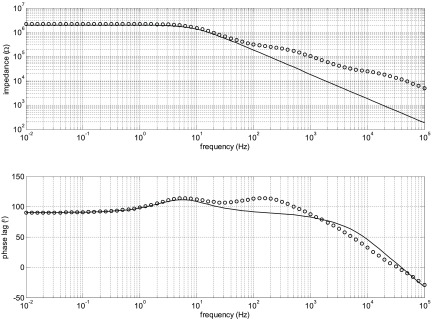
The upper panel is the impedance magnitude of the leakage impedance *Z*_leak_ from the substrate to the PBS. The solid line is 8.6 nF in parallel with 2 MΩ and it causes the peak in phase lag in the EIS seen in the lower panel. If *Z*_leak_ is modelled by three RC pairs in series so that the magnitude changes more slowly (*o*), the effect is to widen the  >90° peak in the measured phase lag (lower panel).

Figure 12 shows a rather constant phase lag over 90° for a wide range of frequency (from 0.1 to 100 Hz). To see how this might happen we tested the effect of two impedances between substrate and depth electrode (*Z*_leak_ in figure 22). The first was 8.6 nF in parallel with a 2 MΩ resistance, imagining that the resistance is a leakage through the encapsulant to the PBS, and 8.6 nF was the measured value. The magnitude of this *Z*_leak_ is shown at the top of figure 23 and it causes a phase spectrum for the measurement in the lower panel: there is a narrow peak with phase lag  >90°.

It is often observed that the electrochemical cells that are leakages (two small electrode areas and a liquid-filled channel) actually have an impedance that has a constant-phase (CP) characteristic which means that the impedance magnitude falls at a rate that is slower than 1 decade/decade. To imitate such an effect, chosen resistor-capacitor pairs were put in series which gave the second curve in the top panel of figure 23. Although a crude approximation to a CP element, the magnitude falls more slowly above the break frequency. The effect of this on the measured impedance is to widen the region over which the phase lag in the spectrum exceeds 90°.

## 6. Discussion

### 6.1. Review of design and development

The development of the apparatus has been an expensive gamble. Practical-sized IDCs are only of the order of 100 pF which is quite a small capacitance. Had the background capacitance of the apparatus been large in comparison, the measurement ceiling would have been lower and consequently the additional multiplexing to accommodate all the samples would have spoilt the performance of the *Modulab*. In fact, with the shielding that we incorporated into the design, the capacitance when the reed relay connects the *Modulab* to the tube connectors, but without a tube present, is only about 4 pF compared to the unconnected cable from the *Modulab* of 0.8 pF. This shows the module adds very little capacitance to the cables.

During development, we have spent some time reducing interference by arranging cable and screening circuits. All the mains cables originate from one connection to the supply, so there are no earth loops, and the LO output from the *Modulab* is not used to earth the screens. USB cables are all in the screened box under the bench but the Ethernet cable (*Modulab* to PC) is not (supplementary figure B). Despite the fact that the lab is in central London, at first floor level next to a busy road, and also close to the mains cables for the building, the *Modulab* can generally extract a satisfactory impedance spectrum despite the noise and interference seen at the femtoammeter output. We take precautions of switching off the room lights and discouraging entry to the lab (which is also Faraday cage) during measurements. The outstanding problem which still sometimes spoils the spectra at the lowest frequencies (and lowest currents) is the occasional current spike, usually of 10 s of pA, such as those shown in figure 11.

We saw management of water as critical to the design. Electrical leakage anywhere along the pathway from the *Modulab* input sockets to the IDCs might spoil the measurements and water, perhaps condensed from vapour evaporating from the hot tank, might cause this leakage. The features of the design that meet this requirement (at least after a year with a tank at 67 °C) are the baffle to reduce vapour escaping from the hot water, ventilation above the baffle and the cleaning and encapsulation of the horizontal boards that support the tube connections (supplementary figure A). As measured day-to-day and shown on the GUI dashboard, the RH above the baffle is generally below 50%. Also important is the design inside the tube and under the bung, where RH  =  100%. Here we rely on the ability of some adhesive silicones to maintain effective glued joints in warm water: the silicone tubes pass right through the bungs so that adhesion to the bung (PEEK) is unnecessary.

### 6.2. Comment on the results

Through comparative tests with samples whereby the on-chip tracks between the IDC and the bonds pads were severed by laser ablation, we have shown that most of the measured capacitance is from the IDC itself (table 3). However, this does not mean that changes in the impedance are necessarily due to encapsulation deterioration on the IDC (e.g. figure 10). The increasing leakage seen at low frequencies could originate somewhere other than the IDC itself, such as the bond pads, at the bung or on the module. This is an important consideration when planning the experiments: how many dummy samples (with no IDC connected) are required? And what measurements should be done as the samples appear to be failing or have failed and are removed from the tank?

The purpose of the apparatus is to observe changes at the IDCs as a result of time, temperature and the aging voltage that stresses the sample. However, it is unavoidable, given the many samples and multiplexing, that from an electrical point of view, the apparatus is much more complicated than an IDC connected to the Modulab. Users of the apparatus need to be able to relate the measured impedance spectra to this complicated structure and we have shown that this can be done using SPICE models. As shown in section 5, these SPICE models can be simplified by lumping together components in parallel and removing components that have little effect, in order to discover what determines how the shape of the spectral changes. We used this approach to understand the high-frequency asymptote, and why the phase lag may exceed 90° due to leakage from the depth electrodes (if floating) to SHIELD or from the substrate to PBS. SPICE simulation also elucidated the advantages of connecting one depth electrode in each tube to LO through a resistor: (i) it prevents leakage from the depth electrode conductors to LO from causing phase lags over 90° (section 5.3); (ii) it raises the measurement ceiling for IDCs with conducting substrates and thin coatings of encapsulant (section 5.4); and (iii) it reduces interference from the aging voltages that are being applied to other tubes in the tank (figure 13). The effect of leakages other than lateral leakage between the combs of the IDC can also be investigated using SPICE, as we have shown in section 5.5. Such a simulation is not a proof of a particular type of failure but it may be helpful in suggesting where to look for encapsulation failure at post-mortem examination, or what electrical tests to do after the sample is removed which might or might not endorse the conjecture about the failure of deceased samples.

### 6.3. What the apparatus can do

We have described an apparatus that allows the Modulab to be used for monitoring many samples over a long period. It is a versatile arrangement as several different aging voltages can be applied, alternating, ‘DC’ or application-specific waveforms. The samples may be in baths set at the same or different temperatures. The effect of different bathing solutions can easily be tested because each sample is in a separate test tube. It is not limited to the IDCs shown in figure 2; we are now also testing IDCs with a smaller conductor-conductor gap and higher electric field strength. These were made in an IC foundry and are being tested with DC bias. Catastrophic failure of a sample is either by short-circuit, open-circuit or a sudden reduction in the capacitance indicating that a track in one metal comb has corroded through. Hence the layout and in particular the choice of capacitance for the IDC, is a compromise between larger values that will allow detection of corrosion of the track that isolates only a small part of one comb, versus smaller values which make the arrangement more sensitive to shunt leakage impedance. The circuit technology is not limited to thin film or CMOS.

The apparatus can be used with small or large batches of samples. Our first experiment has been a materials comparison between three different passivation layers with two difference silicone encapsulants. With batches of 13, we see clear differences that will let us choose the best combination. In experiments in which the life of a device is to be predicted by accelerated aging at two or more elevated temperatures, batch sizes of 50 or more have been used (e.g. Merret [16]), and this apparatus can be configured for such an experiment, but it would not be suitable for the  >600 samples in each batch used by, for example, Nachbauer [17].

In the literature on the reliability of polymer-encapsulated integrated circuits, the reported failures are mostly corrosion of the aluminium (alloy) top metal not involving the deeper active structures which would be p- and n-MOS transistors in CMOS ICs. Nevertheless, one might want to investigate the stability of these transistors in long-term use. At least their leakage properties could be measured with this apparatus, if very large transistors were fabricated, and thus measure either their gate-substrate impedances or the drain-source impedances with the gates connected to the source. The Modulab can produce a wide variety of test voltages including alternating with superimposed bias, which might be appropriate for such transistor tests. At the time of writing we have only done insulation tests which are more fundamental to having a viable implanted device technology.

### 6.4. Using the apparatus in implant life-testing research

Two recent papers should be mentioned on accelerated life testing polymer-encapsulated implants.

Since we started designing the apparatus described in this paper, the team at IIT in Chicago have published the results of an accelerated aging test on their Wireless Floating Microelectrode Array in which an integrated circuit is encapsulated in adhesive silicone rubber. They operated the devices (*n*  =  4) so that they were continuously functioning (i.e. under electrical bias) in an autoclave at 121 °C; none failed in a year [18]. This is a very encouraging result but as the authors comment, it does not let one predict in-body lifetime: more experiments will be needed to do that.

Huang *et al* [19] tested IDCs connected to an ingenious inductively-coupled circuit. These dummy implants used ceramic substrates and gold tracks; these were cleaned in various ways and encapsulated in epoxy. When submerged at 50 °C, they were biased to about 11.5 V across a 25 *µ*m gap continually by magnetic induction from a coil outside the water bath. The test was run for a year and the results reaffirmed the importance of the pre-encapsulation clean. An acceleration factor was assumed for temperature and allowance was made for duty cycle and track length in the implant to arrive at an estimated one-year reliability of 99.97%. Although the resonant frequency changes with time for each sample, corresponding to changes in IDC impedance, there is no analysis of the question: whether the rate of change of this frequency can be used to predict the time to failure.

The use of measurements from IDC samples at elevated temperatures to predict the life of implanted devices in the body is complicated and beyond the scope of this paper. We intend to write a review shortly that will consider the materials, physics and statistical aspects of the problem. In that review we will consider how impedance measurements may reduce the time taken to estimate the implant lifetime.

Osenbach [20] measured time to failure in DC tests at 85 °C and 85%RH on ICs with aluminium metallisation and three different oxy-nitride passivation layers. After wire-bonding, the chips were encapsulated in silicone rubber (‘RTV’). He found that there were three types of failure which, with the technology of the time, occurred at different periods after the start. Early failures (<500 h) were due to inadequate cleaning which caused corrosion at the bond pads. Mid-life failures (500–1500 h) were due to voids in the encapsulant on the surface of the passivation; water condensed into these voids and dissolved the passivation which, on breaking through to the underlying metal caused rapid corrosion. Finally, at  >1500 h, failures occur by channels forming between the tracks which allow voltage-driven electrolysis and corrosion. Osenbach termed the latter a ‘wear-out’ failure, implying that this is the eventual fate of such devices. Of these three types of failure, only the latter is likely to appear as a detectable change in impedance much before the track(s) become significantly corroded.

With this in mind, the apparatus may be used in the following types of experiment.

1. Materials comparisons (see above).
2. To validate the implant technology (IC type, cleaning method, encapsulant, encapsulating method), tests will be done to look for early failures of Osenbach’s first two types. The experiment should be designed to examine the following questions. What fraction of samples fail in this way and over what period? Can these failures be predicted from changes in impedance? Could and should implants have a ‘burn-in’ (active soak test) to screen out incipient failures?
3. For the wear-out process described by Osenbach, an experiment should investigate whether catastrophic failure can be predicted from prior changes in the impedance. If prediction is possible, the impedance data should be analysed to see what parameter(s) best predict time to failure. For the impedance-measurement to be really useful in accelerated testing, the time until actual failure should be substantially longer than the time until the impedance parameter(s) reach threshold.
4. Some implants, or parts of implants, are not exposed to continuous bias, such as those driven by alternating voltages [21]. It may be that changes in impedance are seen, but these do not precede catastrophic failure within a practical period for the experiment. In that case, it may be helpful to choose some parameter as an end-of-life criterion just so that something can be said about the durability of the device; for example, that the impedance at the functional frequency has not declined more than ten-fold. Nachbauer [17] used a CMOS transistor parameter as an indicator of failure and still found an Arrhenius relationship between temperature and time to this parametric failure.

These tests are to be done on IDCs, not active implants, and they are intended to demonstrate the viability of the technology under realistic conditions of bias, duty cycle, etc. There is still an argument for testing actual implants *in vitro* at elevated temperature(s) but such tests are likely to be expensive. The IDC tests might prove the technology before funding manufacture of actual implants and, having found the activation energy for the failure mode(s), may allow implants to be soak tested at only one temperature to confirm their lifetime performance.

## 7. Conclusions

If the possible benefits from ICs & MEMs to implantable medical devices are to be realised, it must be shown that they can be sufficiently reliable. At present, the optimal materials and methods for protecting such devices are not established, and generally insufficient data is available to predict the lifetime in the body under the conditions of use (electrical and mechanical stresses). This paper describes an apparatus that can be used to compare materials and fabrication methods by applying accelerated aging tests with high temperature and voltage stress.

In our design, one *Modulab* with standard leads could conveniently be used with seven water baths which might contain 7  ×  96  =  672 IDC samples. If two modules are used per sample type, which is what we are doing, so 13 or 14 samples per type, then this capacity allows 49 types to be tested simultaneously. The semi-automatic arrangement means that the experiment needs a few minutes of attention each day that measurements are to be performed but this seems a good way to follow the changes that occur without being burdensome. After running the 67 °C bath for over a year, we are confident that at this temperature at least, there are no short-term problems from water harming the instrumentation.

A critical feature of such an apparatus is the measurement ceiling because this and the measurement time determine the smallest void that can be detected (section 2). For IDC on an insulating substrate, ideally the ceiling would only be due to the capacitance between the combs. On a conducting substrate, such as an IC, this will still be the ceiling if the substrate is connected with the shield to LO (earth); we have an alternative arrangement with capacitive coupling from the substrate to the bathing liquid (e.g. PBS) through the thin protective layers, which raises the ceiling (figure 19) but also allows phase lags over 90° if the substrate encapsulation is flawed or damaged (figure 23). The general point is that the IDC that is being measured in a large apparatus with many samples is much more complicated than if it was directly connected to the measuring instrument. Thus it follows that the effect of this larger system on the measured EIS must be understood if the data is to be properly interpreted. With IDCs on conducting substrates, the EIS will show reductions in magnitude and phase at low frequencies if leakages shunt the IDC but the EIS may also show increases in phase lag and impedance magnitude due to leakage from the substrate to the bathing liquid.

In due course, we expect to report results from these experiments, both comparing different materials and investigating acceleration factors. We hope that this paper will encourage workers in other labs to set up dedicated accelerated life-testing apparatus and thus contribute valuable comparative data to our growing field.
